# Rice Phytochrome-Interacting Factor-Like1 (OsPIL1) is involved in the promotion of chlorophyll biosynthesis through feed-forward regulatory loops

**DOI:** 10.1093/jxb/erx231

**Published:** 2017-07-11

**Authors:** Yasuhito Sakuraba, Eun-Young Kim, Su-Hyun Han, Weilan Piao, Gynheung An, Daisuke Todaka, Kazuko Yamaguchi-Shinozaki, Nam-Chon Paek

**Affiliations:** 1 Department of Plant Science, Plant Genomics and Breeding Institute, and Research Institute of Agriculture and Life Sciences, Seoul National University, Seoul, Republic of Korea; 2 Department of Plant Molecular Systems Biotechnology, Crop Biotech Institute, Kyung Hee University, Yongin, Republic of Korea; 3 Graduate School of Agricultural and Life Sciences, The University of Tokyo, Tokyo, Japan

**Keywords:** Chlorophyll biosynthesis, *OsCAO1*, *OsGLK*, *OsPIL1*, *OsPORB*, rice, transcriptional regulation

## Abstract

In phototrophic plants, the highly conserved and tightly regulated process of chlorophyll (Chl) biosynthesis comprises multi-step reactions involving more than 15 enzymes. Since the efficiency of Chl biosynthesis strongly affects plant productivity, understanding the underlying regulatory mechanisms in crop plants can be useful for strategies to increase grain and biomass yields. Here, we show that rice (*Oryza sativa*) Phytochrome-Interacting Factor-Like1 (OsPIL1), a basic helix-loop-helix transcription factor, promotes Chl biosynthesis. The T-DNA insertion knockdown *ospil1* mutant showed a pale-green phenotype when grown in a natural paddy field. Transcriptome analysis revealed that several genes responsible for Chl biosynthesis and photosynthesis were significantly down-regulated in *ospil1* leaves. Using promoter binding and transactivation assays, we found that OsPIL1 binds to the promoters of two Chl biosynthetic genes, *OsPORB* and *OsCAO1*, and promotes their transcription. In addition, OsPIL1 directly up-regulates the expression of two transcription factor genes, *GOLDEN2-LIKE1* (*OsGLK1*) and *OsGLK2*. OsGLK1 and OsGLK2 both bind to the promoters of *OsPORB* and *OsCAO1*, as well as some of genes encoding the light-harvesting complex of photosystems, probably promoting their transcription. Thus, OsPIL1 is involved in the promotion of Chl biosynthesis by up-regulating the transcription of *OsPORB* and *OsCAO1* via trifurcate feed-forward regulatory loops involving two OsGLKs.

## Teaser

In the tightly regulated chlorophyll biosynthesis pathway, the transcription factor Rice Phytochrome-Interacting Factor-Like1 promotes chlorophyll biosynthesis by up-regulating chlorophyll biosynthetic genes through feed-forward regulatory loops involving *GOLDEN2-LIKE1* (*OsGLK1*) and *OsGLK2*.

## Introduction

Chlorophyll (Chl), a green pigment found in phototrophic organisms, harvests light and transfers the resulting excitation energy to other components of the electron transport chain. Land plants, green algae, and a few cyanobacteria synthesize and use two types of Chl, Chl *a* and Chl *b* (Melis, 1991; Blankenship, 1992). In addition to its light-harvesting role, Chl and its intermediates also act as strong photosensitizers and generate reactive oxygen species when they are present in excess and irradiated by light (Meskauskiene *et al.*, 2001; Nagata *et al.*, 2005; Skribanek *et al.*, 2008; Hideg *et al.*, 2010). To prevent adverse effects, Chl biosynthesis is a highly co-ordinated process, with multi-step reactions catalysed by various biosynthetic enzymes. In *Arabidopsis thaliana*, 15 enzymes associated with Chl biosynthesis have been identified to date via several biochemical and genetic approaches, as well as genomic analysis (Tanaka and Tanaka, 2007). Since mutations in some Chl biosynthetic genes negatively affect Chl biosynthesis and accumulation, arabidopsis mutants of some of these genes show a pale-green phenotype (Oster *et al.*, 2000; Mochizuki *et al.*, 2001; Nagata *et al.*, 2005). Chl levels are largely controlled by the balance between anabolism and catabolism (Hörtensteiner and Kräutler, 2011; Czarnecki and Grimm, 2012), which has a direct effect on photosynthetic efficiency, a process that greatly affects productivity in cereal crops.

Rice (*Oryza sativa*), a major cereal crop worldwide, has been intensively studied as a model monocot species. The regulatory mechanism underlying Chl biosynthesis has been explored in rice using Chl-deficient mutants, such as pale-green, variegated, or albino mutants. Among Chl-deficient rice mutants, several are associated with Chl biosynthetic enzymes. For example, the *chlorina1* (*chl1*) and *chl9* mutants affect the genes encoding OsCHLD and OsCHLI, two of the three subunits of Mg-chelatase; the *chl1* and *chl9* mutants have yellowish-green leaves due to reduced Mg-protoporphyrin IX and total Chl contents (Zhang *et al.*, 2006). Chlorophyllide *a* oxygenase (CAO) and 3,8-divinyl protochlorophyllide *a* 8-vinyl reductase (DVR) are key enzymes involved in Chl homeostasis (Tanaka *et al.*, 1998; Nagata *et al.*, 2005), and rice mutants of *OsCAO1* and *OsDVR* also have a pale-green leaf phenotype (Lee *et al.*, 2005; Wang *et al.*, 2010). By contrast, the rice mutant *faded green leaf* (*fgl*), harboring a mutation in *OsPORB* (encoding protochlorophyllide *a* oxidoreductase B), has variegated leaves, especially under high-light conditions (Sakuraba *et al.*, 2013).

In addition to mutants of Chl biosynthetic enzymes, other Chl-deficient rice mutants have also been reported. The *young leaf chlorosis1* (*ylc1*) mutant, with a mutation in a DUF3353 superfamily gene, has pale-green leaves at the early seedling stage (Zhou *et al.*, 2013). The *yellow green leaf2* (*ygl2*) mutant, which is impaired in heme oxygenase 1 (HO1) production, also has pale-green leaves, indicating that HO1 indirectly affects Chl biosynthesis, as heme and chlorophyll share the same substrates prior to protoporphyrin IX formation (Chen *et al.*, 2013).

GOLDEN2-LIKE (GLK) is another important factor that helps to regulate Chl homeostasis. *GLK* genes encode GARP-type transcription factors (TFs) that play regulatory roles in chloroplast development and Chl biosynthesis, and thus *glk* mutants in various plants, including arabidopsis, rice, tomato, and the moss *Physcomitrella patens*, have pale-green leaves (Fitter *et al.*, 2002; Yasumura *et al.*, 2005; Powell *et al.*, 2012; Wang *et al.*, 2013). In arabidopsis, two GLK proteins, AtGLK1 and AtGLK2, bind to the promoters of genes encoding components of the photosynthetic apparatus, including the light-harvesting complex of photosystem II (LHCII), as well as Chl biosynthetic genes, including *CAO*, *PORB*, and *CHLH*, and up-regulate their expression (Waters *et al.*, 2009). However, it is still unknown whether two GLK proteins in rice, OsGLK1 and OsGLK2, also directly up-regulate the expression of genes for photosynthetic apparatus components and Chl biosynthesis, although the *osglk1 osglk2* double-mutant has pale-green leaves due to reduced Chl accumulation throughout development (Wang *et al.*, 2013).

Phytochrome-Interacting Factors (PIFs) are plant-specific basic helix-loop-helix (bHLH)-type TFs whose regulatory roles have been widely studied in arabidopsis (Castillon *et al.*, 2007). PIF TFs regulate various biological processes in a red-light phytochrome (phy)-dependent manner, including seed germination (Oh *et al.*, 2004), hypocotyl elongation (Nusinow *et al.*, 2011), flowering (Kumar *et al.*, 2012), leaf senescence (Sakuraba *et al.*, 2014), Chl biosynthesis (Huq *et al.*, 2004), and the biosynthesis or signaling pathways of phytohormones, including gibberellic acid (Feng *et al.*, 2008), auxin (Franklin *et al.*, 2011; Oh *et al.*, 2014), and brassinosteroids (Shahnejat-Bushehri *et al.*, 2016). However, to date, the regulatory roles of rice PIFs are largely unknown. Six rice PIF TFs, termed OsPIF-Like11 (OsPIL11), OsPIL12, OsPIL13 (also termed OsPIL1), OsPIL14, OsPIL15, and OsPIL16, are considered homologs of arabidopsis PIF TFs based on sequence similarity (Nakamura *et al.*, 2007). *OsPIL15*-overexpressing (OX) transgenic rice plants exhibit shorter shoots and roots under dark conditions, indicating that OsPIL15 is involved in growth of etiolated seedlings (Zhou *et al.*, 2014), similar to arabidopsis PIFs (Shin *et al.*, 2009). Microarray analysis has shown that *OsPIL1*/*OsPIL13* (hereafter referred to as *OsPIL1*) is a stress-responsive gene (Maruyama *et al.*, 2012). Todaka *et al.* (2012) reported that overexpressing *OsPIL1* promotes internode elongation by increasing internode cell size, especially under drought-stress conditions. Therefore, *OsPIL1*-OX plants are significantly taller than the wild-type, but transgenic rice plants expressing *OsPIL1*-RD (fused to a transcriptional repression domain) are shorter (Todaka *et al.*, 2012).

In this study, we found that OsPIL1 is a key regulator of Chl biosynthesis. The T-DNA insertion *ospil1* knockdown mutant exhibited a pale-green leaf phenotype, with significantly reduced levels of Chl and Chl-binding proteins compared to the wild-type. Microarray analysis revealed that the genes for Chl biosynthetic enzymes and the photosynthetic apparatus, as well as two *OsGLK* genes, were significantly down-regulated in *ospil1* mutants. Furthermore, promoter binding and transactivation assays revealed that OsPIL1 binds to the promoters of *OsPORB*, *OsCAO1*, *OsGLK1*, and *OsGLK2* and up-regulates their expression. Moreover, OsGLK1 and OsGLK2 bind to the promoters of *OsPORB* and *OsCAO1*. We propose a possible model for the regulation of Chl biosynthesis in rice via OsPIL1.

## Materials and methods

### Plant material and growth conditions

A T-DNA insertion knockdown mutant of *OsPIL1* (LOC_Os03g56950; PFG_4A-03590.R; hereafter termed *ospil1*) was isolated in the Korean *japonica* rice cultivar ‘Dongjin’ (hereafter referred to as the wild-type; WT) using information obtained from the Salk Institute Genomic Analysis Laboratory (http://signal.salk.edu/cgi-bin/RiceGE) (Jeong *et al.*, 2002). The plants were grown in a paddy field at the Seoul National University Experiment Farm under natural long-day (NLD) conditions (latitude 37° N, Suwon, Korea). The seeds were sown on seedbeds in a greenhouse and after 1 month the seedlings were transplanted to the paddy field. Rice plants were also grown in growth chambers under short-day (SD; 10 h light, 30 °C / 14 h dark, 24 °C) and long-day (LD; 14.5 h light, 30 °C / 9.5 h dark, 24 °C) conditions using light-emitting diodes at a photon flux density of approximately 300 μmol m^–2^ s^–1^ PAR, with 60% relative humidity.

### Plasmid construction and plant transformation

The *OsPIL1* cDNA was amplified by RT-PCR using the gene-specific primers OsPIL1-F and OsPIL1-R (Supplementary Table S1 at *JXB* online) and sub-cloned into the pCR8/GW/TOPO vector (Invitrogen). After verifying its sequence, the *OsPIL1* cDNA was inserted into the pMDC32 Gateway binary vector containing the 35S promoter (Curtis and Grossniklaus, 2003) through LR recombination (Lambda integrase/excisionase, Elpisbio, Korea). The resulting plasmid was transformed into *Agrobacterium tumefaciens* strain EHA105, which was introduced into rice calli from mature *ospil1* embryos using *Agrobacterium*-mediated transformation (Jeon *et al.*, 1999; Lee *et al.*, 2006). The transgenic rice plants were selected on 2N6 medium containing hygromycin (50 mg l^–1^) and confirmed by genomic PCR using specific primers (see Supplementary Table S1).

### RNA extraction, reverse transcription (RT), and quantitative PCR (qPCR) analysis

Total RNA was extracted from leaf tissues using an MG Total RNA Extraction kit (Macrogen, Korea) according to the manufacturer’s instructions. First-strand cDNA for RT was synthesized from 2.5 µg total RNA using the oligo(dT)_15_ primer and M-MLV reverse transcriptase (Promega) and diluted with water to 100 µl. The relative expression levels of *OsPIL1* and Chl biosynthetic genes were measured by RT-qPCR using gene-specific primers and either *Ubiquitin5* (*UBQ5*; Os01g0328400) or *GAPDH* (glyceraldehyde-3-phosphate dehydrogenase; Os06g0666600) as an internal control (see Supplementary Table S1) (Jain *et al.*, 2006), along with GoTaq qPCR Master Mix (Promega) in a total reaction volume of 20 μl. The expression level of each gene was measured by the relative quantification method using the LightCycler 480 real-time PCR system (Roche Applied Science) under the following cycling conditions: 95 °C for 2 min, followed by 45 cycles of 95 °C for 10 s, and 60 °C for 1 min.

### Quantification of photosynthetic pigments

To measure total chlorophyll (Chl) and carotenoid (Car) contents, pigments were extracted from leaf tissues using 80% ice-cold acetone. Chl and Car concentrations were determined by spectrophotometry as described previously (Porra *et al.*, 1989).

### SDS-PAGE and immunoblot analysis

To extract total proteins, the middle parts of the first leaves in the main culms of 6-week-old rice plants grown under LD conditions (14 h light/10 h dark) were used. To extract total proteins, leaf tissues were ground in liquid nitrogen and 10-mg aliquots were homogenized with 100 µl of sample buffer [50 mM Tris, pH 6.8, 2 mM EDTA, 10% glycerol, 2% sodium dodecyl sulfate (SDS), and 6% 2-mercaptoethanol]. The homogenates were centrifuged at 10000 *g* for 3 min, and the supernatants were denatured at 80 °C for 5 min. A 4-µl aliquot of each sample was subjected to 12% (w/v) polyacrylamide SDS-polyacrylamide gel electrophoresis (PAGE), and the resolved proteins were electroblotted onto a Hybond-P membrane (GE Healthcare, USA). Antibodies against photosystem proteins Lhca1, Lhca2, Lhcb1, Lhcb2, Lhcb4, CP43, and PsaA (Agrisera, Sweden) were used for immunoblot analysis. The level of each protein was measured using the ECL system with WESTSAVE (AbFrontier, Korea) according to the manufacturer’s protocol.

### Transmission electron microscopy

Transmission electron microscopy was performed using a previously described method (Inada *et al.*, 1998) with some modifications. The middle part of the first leaf in the main culm was used for the experiments. Small leaf pieces were fixed in modified Karnovsky’s fixative (2% paraformaldehyde, 2% glutaraldehyde, and 50 mM sodium cacodylate buffer, pH 7.2), followed by three washes with 50 mM sodium cacodylate buffer, pH 7.2 at 4 °C for 10 min. The samples were post-fixed at 4 °C for 2 h with 1% osmium tetroxide in 50 mM sodium cacodylate buffer, pH 7.2, and washed twice with distilled water at room temperature. The samples were stained *en bloc* in 0.5% uranyl acetate at 4 °C overnight and dehydrated in an ethanol gradient solution with propylene oxide, followed by infiltration with Spurr’s resin. The samples were polymerized at 70 °C for 24 h and sectioned with an ultramicrotome (MT-X). The sections were mounted on copper grids and stained with 2% uranyl acetate for 7 min and with Reynolds’ lead citrate for 7 min. Micrographs were obtained with a LIBRA 120 transmission electron microscope.

### Yeast one-hybrid assays

Yeast one-hybrid assays were performed according to the Yeast Protocols Handbook (Clontech). *OsPIL1* cDNA was inserted into the pGAD424 vector (Clontech) as prey. DNA fragments corresponding to the promoters (1050 bp) of *OsPORA*, *OsPORB*, and *OsCAO1* were cloned into the pLacZi vector (Clontech) as bait. For each gene, two DNA fragments (–2000 to –951, and –1050 to –1 from the start codon) were prepared. Primers used for cloning are listed in Supplementary Table S1. The yeast strain YM4271 was used for the bait and prey clones, and β-galactosidase activity was measured by liquid assay using chlorophenol red-β-D-galactopyranoside (CPRG; Roche Biochemicals).

### Microarray analysis

Three-week-old WT and *ospil1* plants grown under LD conditions were used for microarray analysis. Total RNA was extracted from the first leaves of WT and *ospil1* plants using an MG Total RNA Extraction kit according to the manufacturer’s protocol (Macrogen, Korea). Total RNA quality was checked using a 2100 Bioanalyzer (Agilent Technologies). All microarray experiments, including data analysis, were performed according to the manufacturer’s manual (http://www.genomics.agilent.com/literature.jsp?crumbAction=push&tabId=AG-PR-1001&contentType=User+Manual). The arrays were air-dried and scanned using a high-resolution array scanner (Agilent) with the appropriate settings for two-color gene expression arrays. GeneSpring GX 7.3 (Agilent) was used to calculate the intensity ratio and fold-changes, and quantified with the Feature Extraction Software (Agilent). For evaluating the statistical significance and obtaining the *P*-value, one-sample *t*-tests were performed using GeneSpring GX 7.3 (Agilent). Microarray analysis was performed with two experimental replicates with two different biological replicates of WT and *ospil1* samples. Information about phytohormone- and photosynthesis-associated genes was obtained from the Oryzabase (www.shigen.nig.ac.jp/rice/oryzabase).

### Chromatin immunoprecipitation (ChIP) assay

For the ChIP assay, the *35S:OsPIL1-GFP*, *35S:OsGLK1-GFP*, and *35S:OsGLK2-GFP* constructs in the pMDC43 binary vector (Curtis and Grossniklus, 2003) were transfected into rice protoplasts as previously described (Zhang *et al.*, 2011). The protoplasts were then subjected to cross-linking for 20 min with 1% formaldehyde under vacuum. The chromatin complexes were isolated and sonicated as previously described (Saleh *et al.*, 2008) with slight modifications. An anti-GFP antibody (Abcam) and Protein A agarose/salmon sperm DNA (Millipore) were used for immunoprecipitation. After reverse cross-linking and protein digestion, the DNA was purified using a QIAquick PCR Purification kit (Qiagen). The primer sequences for each gene are listed in Supplementary Table S1.

## Results

### *Phenotypic characterization of the* ospil1 *knockdown mutant*

**Fig. 1.**
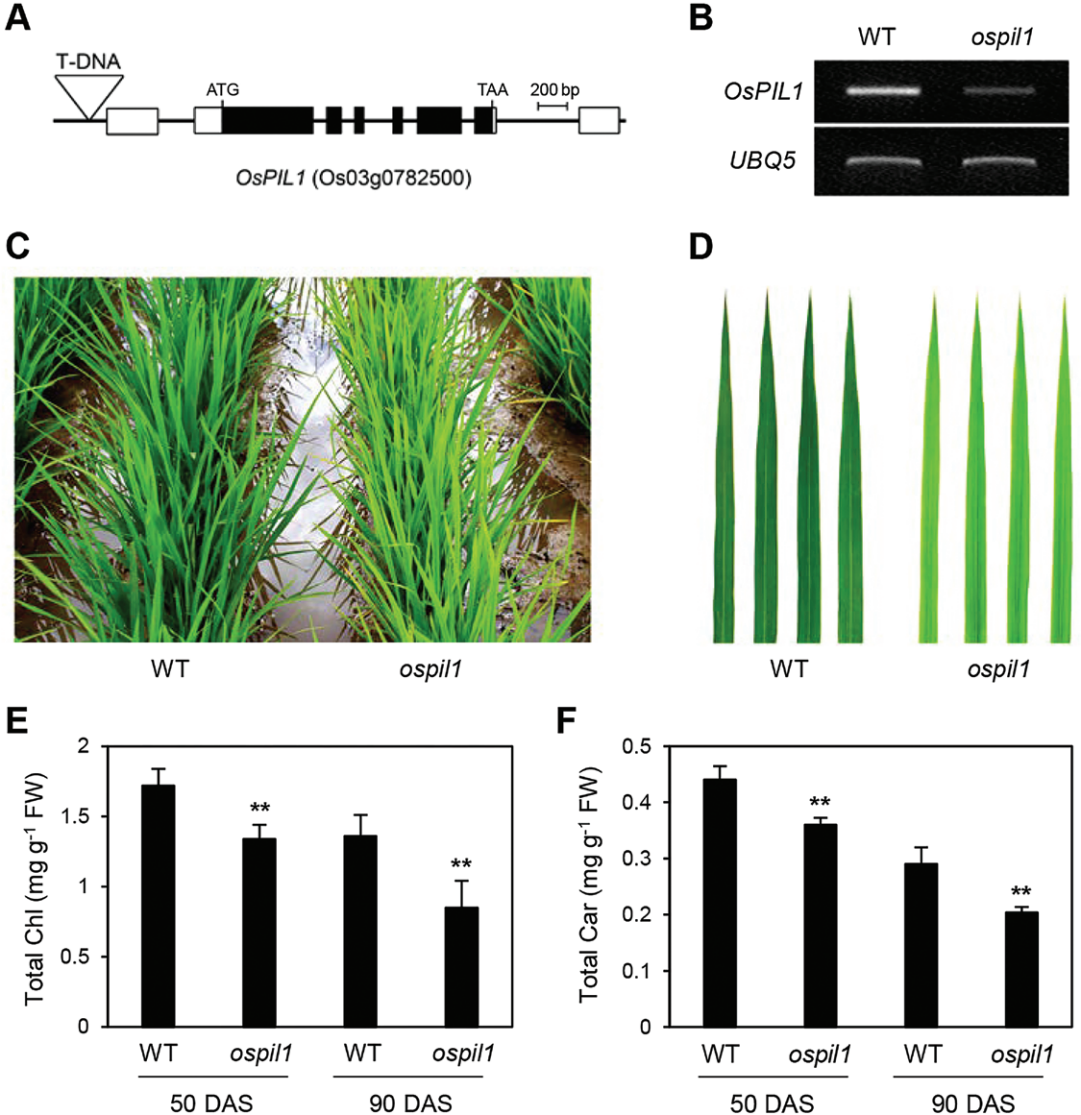
Pale-green leaf phenotype of *ospil1* mutants grown in a paddy field. (A) Gene structure and T-DNA insertion site (inverted triangle) in the 1000-bp upstream region of *OsPIL1* (PFG_4A-03590.R). (B) Decrease in *OsPIL1* transcript levels in the *ospil1* mutant confirmed by RT-PCR. *UBQ5* was used for an internal control. (C, D) Color difference in whole plants (C) and the first leaf of the main culm (D) between WT and *ospil1* at 70 d after sowing (DAS). (E, F) Reduced levels of total Chl (E) and Car (F) in *ospil1*. The first leaves of the main culm at 50 and 90 DAS were used for analysis. Means and SD were obtained from 10 biological replicates. Significant differences between WT and *ospil1* was determined by Student’s *t*-test (** *P*<0.01).

Among rice phytochrome-interacting factors (PIFs), PIF-LIKE1 (OsPIL1; also known as OsPIL13; Os03g0782500) has high similarity to the arabidopsis PIF4 and PIF5 TFs, which play regulatory roles in plant growth and development. OsPIL1 is involved in shoot elongation: *OsPIL1*-overexpressing (*OsPIL1*-OX) rice is taller than its parental cultivar (Todaka *et al.*, 2012). To identify other possible function(s) of OsPIL1, we searched for mutant lines in the RiceGE database (http://signal.salk.edu/cgi-bin/RiceGE) and found one T-DNA insertion line (PFG_4A-03590.R), which harbors a T-DNA fragment in the promoter region of *OsPIL1* (Fig. 1A). Using RT-qPCR analysis, we confirmed that this line has much lower levels of *OsPIL1* transcript than its wild-type parental line, *japonica* cultivar ‘Dongjin’ (hereafter WT; Fig. 1B), indicating that this line is a knockdown mutant of *OsPIL1* (hereafter *ospil1*).

To examine the possible phenotypic effect of the *ospil1* mutation, we grew *ospil1* plants in a paddy field under natural long-day (NLD) conditions (>14 h light/day at 37° N latitude, Suwon, Korea). The *ospil1* leaf blades exhibited a pale-green phenotype compared to the WT (Fig. 1C, D). To verify this phenotype, we measured the contents of photosynthetic pigments at two different developmental stages. At 50 d after sowing (DAS), the Chl contents in *ospil1* leaves were reduced by 16.2% compared to the WT, while those of carotenoids (Car) were reduced by 10.9%. The phenotype was more severe at 90 DAS, with Chl and Car contents in *ospil1* leaves reduced to 24.6% and 18.7% of the WT levels, respectively (Fig. 1E, F).

To confirm that the knockdown mutation in *OsPIL1* is responsible for the pale-green leaf phenotype, we performed a complementation test of the *ospil1* mutant. Using *Agrobacterium*-mediated transformation, we obtained three independent transgenic lines containing *35S:OsPIL1* cDNA, which had normal green leaves throughout development (see Supplementary Fig. S1A, B), and validated the overexpression of *OsPIL1* transcripts in the leaves of transgenic lines by RT-qPCR (Fig. S1C). Consistent with the visible phenotype, total Chl levels in the transgenic lines were similar to those of WT (Supplementary Fig. S1D), confirming that the knockdown mutation in *OsPIL1* results in the development of pale-green leaves.

To examine the pale-green phenotype of *ospil1* leaves in more detail, we grew the plants in a growth chamber under LD conditions (14.5 h light, 30 °C / 9.5 h dark, 24 °C). Similar to the phenotype under NLD conditions, young leaves of 2-week-old *ospil1* plants were pale green (see Supplementary Fig. S2A, B), with lower levels of Chl and Car compared to the WT (Fig. S2C, D). Immunoblot analysis showed that the levels of photosystem proteins were significantly reduced in *ospil1* leaves, with up to a 20–30 % reduction in the levels of light-harvesting complex of photosystem II (LHC II) subunits (Lhcb1, Lhcb2, and Lhcb4), LHC I subunits (Lhca1 and Lhca2), and core subunits of PSII (PsbC) and PSI (PsaA) compared to the WT (Supplementary Fig. S2E). In addition, the *ospil1* mutant displayed a slightly higher Chl *a*/*b* ratio than the WT (Fig. S2F). We also found by transmission electron microscopy analysis that the chloroplasts of the *ospil1* mutant were not defective, but appeared to be slightly smaller and have a looser grana structure compared with the WT (Supplementary Fig. S3).

The chlorosis and/or necrosis observed in some leaf-color mutants in rice largely depend on the photoperiod (Kusumi *et al.*, 2000; Han *et al.*, 2012). Thus, we examined the leaf color of *ospil1* plants grown under SD conditions (10 h light/14 h dark), finding that the leaves of this mutant were paler than those of the WT (see Supplementary Fig. S4A), with lower levels of photosynthetic pigments (Fig. S4B, C). This result indicates that the levels of photosynthetic pigments in *ospil1* are reduced regardless of photoperiod.

### *Mutation of* OsPIL1 *decreases agronomic performance in rice*

**Fig. 2.**
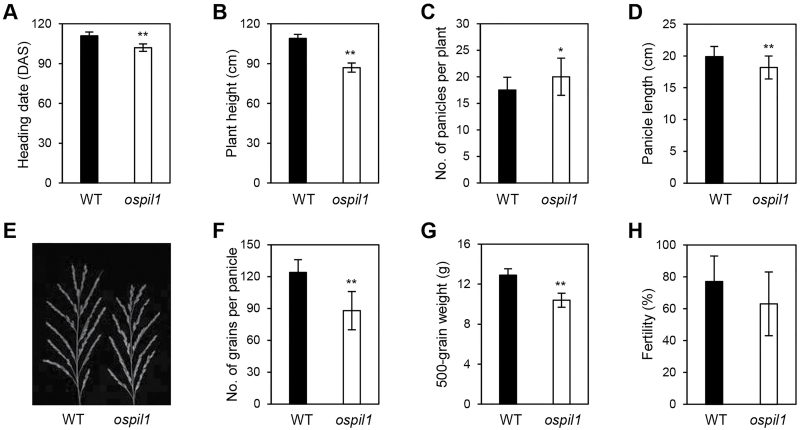
Agronomic traits of *ospil1* plants. (A) Heading date, (B) plant height, (C) number of panicles per plant, (D) panicle length, (E) panicle phenotype, (F) number of grains per panicle, (G) 500-grain weight, and (H) fertility in WT and *ospil1*. Means and SD were obtained from at least 10 biological replicates. Significant differences were determined by Student’s *t*-test (* *P*<0.05, ** *P*<0.01).

Since reduced photosynthetic pigment levels and photosynthetic activity negatively affect plant production, many leaf-color-associated mutants show poor agronomic traits compared to the WT (Sakuraba *et al.*, 2013). To examine the relationship between the mutation in *ospil1* and crop production, we evaluated several agronomic traits in this mutant, including heading date, plant height, panicle length, number of panicles per plant, number of grains per panicle, spikelet fertility, and 500-grain weight under NLD conditions (Fig. 2). The *ospil1* mutant [105 d to heading([DTH)] flowered earlier than the WT (115 DTH) (Fig. 2A). The height of *ospil1* plants was significantly smaller than that of the WT (Fig. 2B), which corresponds to the previous finding (Todaka *et al.*, 2012) that *OsPIL1*-OX plants have significantly increased height due to elongated internode cells. The number of panicles per plant (Fig. 2C) was higher in *ospil1* than in the WT; however, the *ospil1* mutant had significantly lower values for other agronomic traits compared to the WT, including panicle length (Fig. 2D, E), the number of grains per panicle (Fig. 2F) and 500-grain weight (Fig. 2G), without affecting seed fertility (Fig. 2H). These results indicate that the *ospil1* mutation has negative effects on agronomic traits, ultimately reducing rice grain production.

### OsPIL1 directly activates the transcription of Chl biosynthetic genes

To examine the downstream regulatory cascade of OsPIL1, we conducted a genome-wide microarray analysis to identify differentially expressed genes between the WT and *ospil1* in 1-month-old plants under LD conditions. We identified 725 genes that were significantly up-regulated (*ospil1*/WT, >2-fold) and 840 genes (including *OsPIL1*) that were significantly down-regulated (*ospil1*/WT, <2-fold) in *ospil1* compared to the WT. To assess the quality of the microarray data, we investigated whether cell wall-related genes that are up-regulated in *OsPIL1*-OX plants (Todaka *et al.*, 2012) are differentially expressed in *ospil1*. In contrast to their expression patterns in *OsPIL1*-OX, some cell wall-related genes, including expansin, cellulose synthase, and pectinesterase genes, were down-regulated in *ospil1* (see Supplementary Fig. S5). In addition, several genes related to the biosynthesis and signaling pathways of growth-promoting phytohormones were also down-regulated (Supplementary Fig. S6), perhaps leading to the reduced plant height of the mutant (Fig. 2B).

**Fig. 3.**
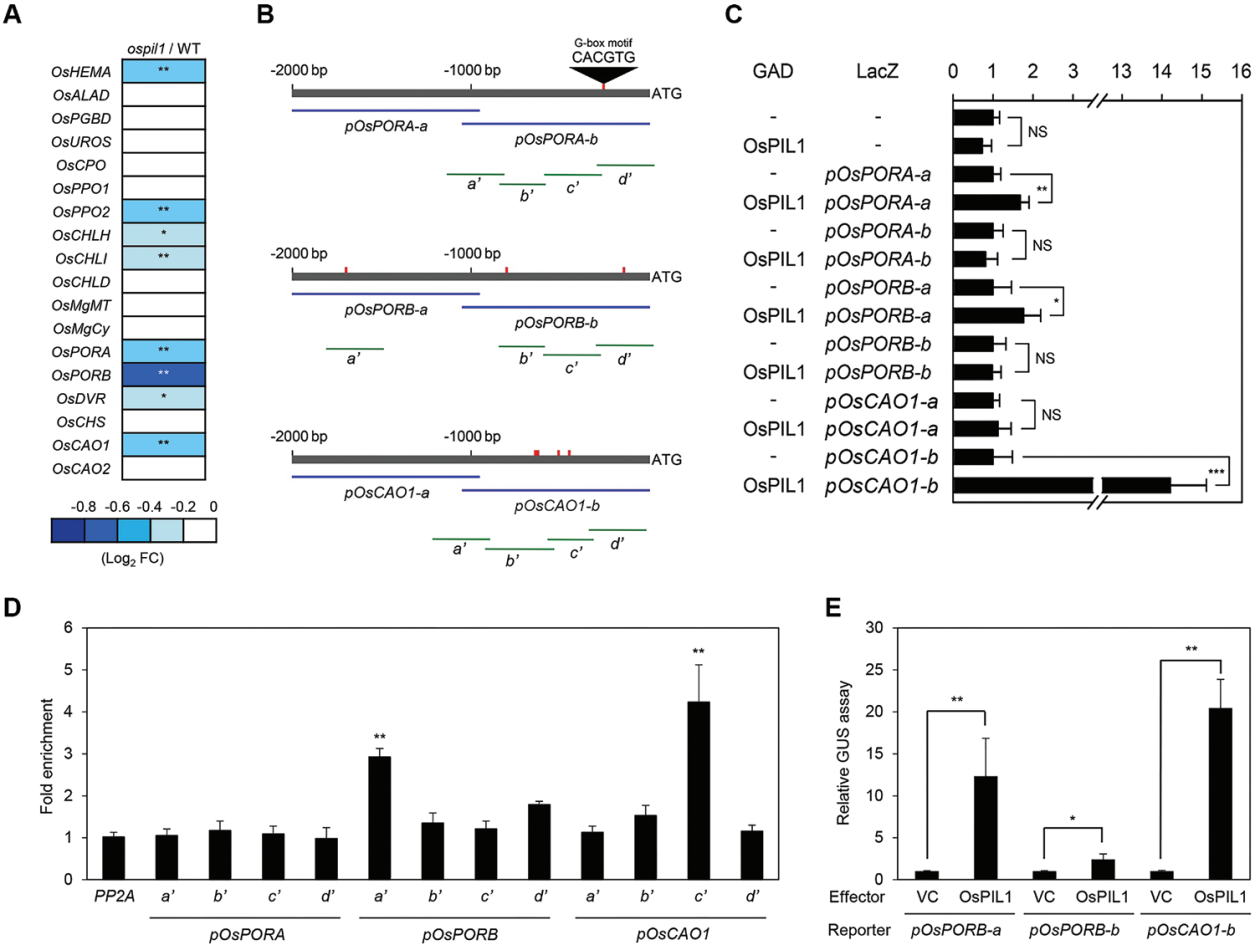
OsPIL1 directly up-regulates *OsPORB* and *OsCAO1* transcription. (A) Relative expression (*ospil1*/WT) of Chl biosynthetic genes. Relative expression levels of genes in *ospil1* were normalized to those of the WT. Asterisks indicate significant difference between WT and *ospil1* plants (* *P*<0.05, ** *P*<0.01). (B) The positions of G-boxes in the promoters of *OsPORA*, *OsPORB*, and *OsCAO1* (–2000 bp to ATG) and the promoter fragments used for the yeast one-hybrid assay (Y1H), transactivation assays (blue horizontal lines), and ChIP assays (green horizontal lines). (C) The binding activity of OsPIL1 to the promoter regions of *OsPORA* (*pOsPORA-a* and *pOsPORA-b*), *OsPORB* (*pOsPORB-a* and *pOsPORB-b*), and *OsCAO1* (*pOsCAO1-a* and *pOsCAO1-b*) examined by Y1H assays. Empty bait and prey plasmids (-) were used for the negative controls. The relative β-galactosidase activity was obtained by normalizing to the level of each negative control. Means and SD were obtained from more than five independent colonies. (D) OsPIL1 binding affinity to the promoter regions of *OsPORA*, *OsPORB*, and *OsCAO1 in planta* examined by ChIP assays. OsPIL1-GFP was transiently expressed in protoplasts isolated from 10-d-old WT seedlings. Fold-enrichment of the promoter fragments was measured by immunoprecipitation with an anti-GFP antibody (see Methods). *PP2A* was used as a negative control. (E) Transactivation of *OsPORB* and *OsCAO1* by OsPIL1. The protoplasts were co-transfected with 5 μl of effector plasmid containing *35S:OsPIL1-GFP* and 3 μl of reporter plasmids containing *pOsPORB-a::GUS*, *pOsPORB-b::GUS*, and *pOsCAO1-b::GUS*. Empty vector was used as a vector control for the effector. Significant differences were determined by Student’s *t*-test (* *P*<0.05, ** *P*<0.01, *** *P*<0.001, NS, not significant).

Nearly 20 enzymes are involved in the biosynthesis of Chl from glutamic acid (Tanaka and Tanaka, 2006), and rice mutants of Chl anabolic enzymes exhibit leaf chlorosis or necrosis (Lee *et al.*, 2005; Zhang *et al.*, 2006; Wang *et al.*, 2010; Sakuraba *et al.*, 2013). Thus, we examined our microarray data to determine whether the genes for Chl biosynthetic enzymes are differentially expressed in *ospil1*. Among the 18 Chl biosynthetic genes, several genes such as *OsHEMA*, *OsCHLH*, *OsPORA*, *OsPORB*, *OsDVR*, and *OsCAO1* were down-regulated in the *ospil1* mutants (Fig. 3A); the down-regulation of these genes in *ospil1* was further confirmed by RT-qPCR analysis (Supplementary Fig. S7). Furthermore, *OsHEMA*, *OsPORA*, *OsPORB*, and *OsCAO1* were up-regulated in *OsPIL1*-OX plants, whereas the expression levels of *OsCHLH* and *OsDVR* were not significantly altered (Supplementary Fig. S8).

Based on the microarray and RT-qPCR analyses described above, it appears that *OsHEMA*, *OsPORA*, *OsPORB*, and *OsCAO1* might be direct targets of OsPIL1. Since OsPIL1, as well as other PIF TFs, specifically bind to the G-box motif (CACGTG) in the promoters of target genes (Todaka *et al.*, 2012), we searched for G-box elements in 2000-bp upstream regions (–2000 bp) of the target genes and found that the promoter regions of *OsPORA*, *OsPORB*, and *OsCAO1* each contain more than one G-box sequence (Fig. 3B). Therefore, we examined whether OsPIL1 directly binds to the promoter regions of these three candidate genes by yeast one-hybrid (Y1H) assays. OsPIL1 bound to the promoters of *OsPORA-a* (–2000 to –1000 bp), *OsPORB-a* (–2000 to –1000 bp), and *OsCAO1-b* (–1000 bp to 0 bp) in the Y1H assay (Fig. 3C), although *OsPORA-a* does not contain a G-box motif. To confirm these interactions *in vivo*, we performed chromatin immunoprecipitation (ChIP) assays using WT protoplasts in which *OsPIL1-GFP* was transiently expressed. Consistent with the results of the Y1H assay, OsPIL1 strongly bound to amplicon-a′ of the *OsPORB* promoter and amplicon-c′ of the *OsCAO1* promoter containing the G-box motif (Fig. 3D). By contrast, OsPIL1 did not bind to the promoter of *OsPORA in vivo*.

To further investigate whether OsPIL1 acts as a transcriptional activator of *OsPORB* and *OsCAO1*, we performed transactivation assays using rice protoplasts. Protoplasts were isolated from the shoots of 10-d-old seedlings and transfected with a plasmid containing *35S:OsPIL1-GFP*, together with plasmids containing the *GUS* reporter gene behind the promoter regions of *OsPORB* (–2000 bp to –1000 bp) and *OsCAO1* (–1000 bp to –1 bp). *OsCAO1* promoter-directed and *OsPORB* promoter-directed GUS activity were significantly enhanced in the cells expressing *OsPIL1*, compared with the vector control (Fig. 3E). Taken together, these results indicate that *OsPORB* and *OsCAO1* are the direct target genes of OsPIL1 among genes encoding Chl biosynthetic enzymes.

### *OsPIL1 directly up-regulates the expression of two* OsGLK *genes*

**Fig. 4.**
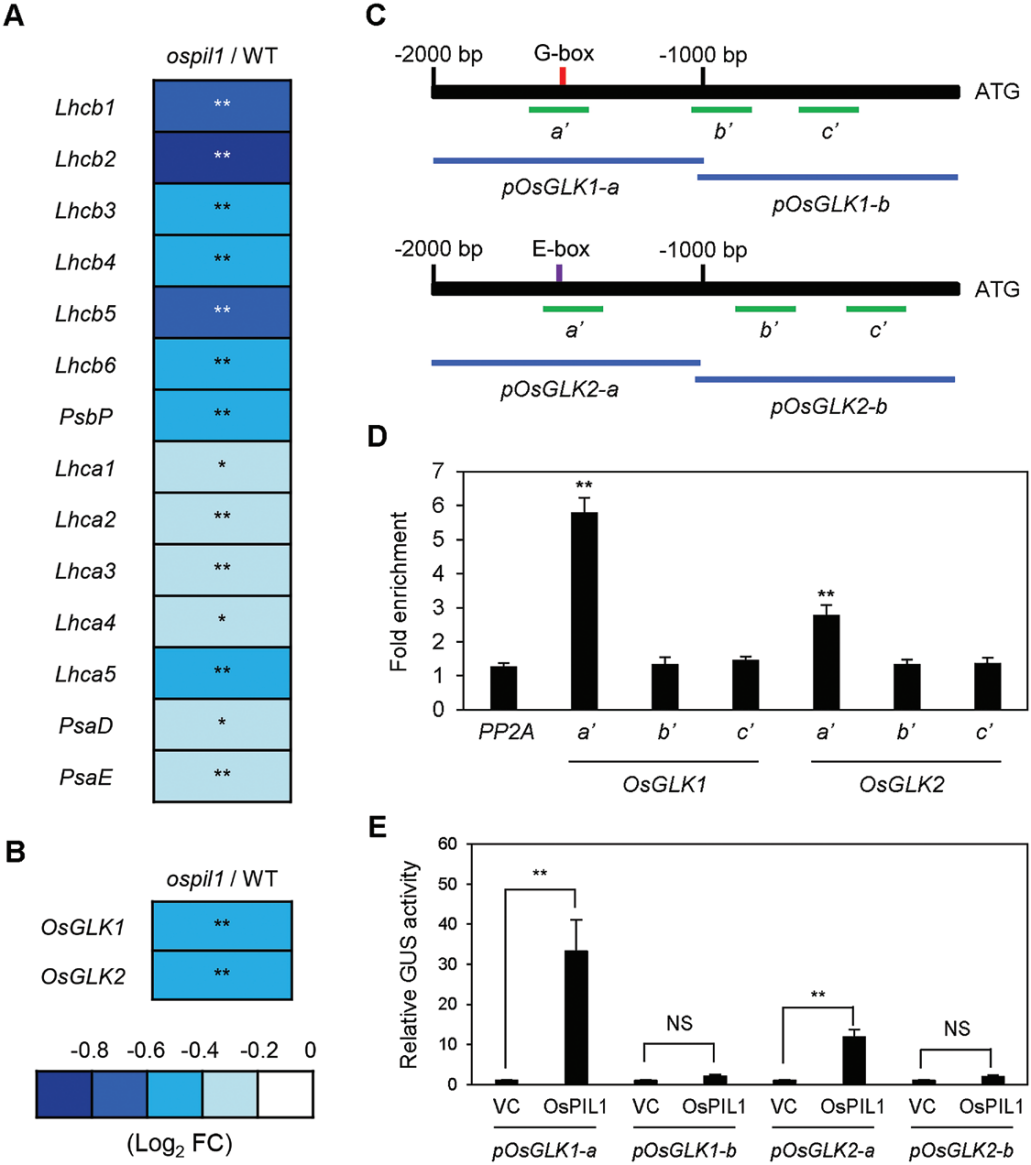
OsPIL1 directly promotes the expression of *OsGLK1* and *OsGLK2*. (A, B) Relative expression levels (*ospil1*/WT) of photosynthetic apparatus genes (A) and two *GLK* genes (B). Relative expression levels of genes in *ospil1* are normalized to those of the WT. Asterisks indicate significant difference between the WT and *ospil1* plants (Student’s *t*-test, * *P*<0.05, ** *P*<0.01). (C) Positions of the G-box (red vertical bar) and E-box (purple vertical bar) in the *OsGLK1* and *OsGLK2* promoters and the promoter fragments (green horizontal bars) used for ChIP and transactivation assays. (D) The binding affinity of OsPIL1 to the promoter regions of *OsGLK1* and *OsGLK2 in planta* examined by ChIP assay. OsPIL1-GFP was transiently expressed in protoplasts isolated from 10-d-old WT seedlings. Fold-enrichment of the promoter fragments was measured by immunoprecipitation with anti-GFP antibody (see Methods). *PP2A* was used as a negative control. (E) Transactivation of *OsGLK1* and *OsGLK2* by OsPIL1. The protoplasts were co-transfected with 5 μl of effector plasmid containing *35S:OsPIL1-GFP* and 3 μl of reporter plasmids containing *pOsGLK1-a::GUS*, *pOsGLK1-b::GUS*, and *pOsGLK2-a::GUS*, and *pOsGLK2-b::GUS*. Empty vector was used as a vector control for the effector. Significant differences were determined by Student’s *t*-test (* *P*<0.05, ** *P*<0.01, NS, not significant).

In the microarray analysis, we also found that genes associated with photosynthesis, such as genes encoding light-harvesting complex subunits of photosystem I and II (*Lhca* and *Lhcb*) and the photosystem I core complex (*PsaD*, *PsaE*, and *PsbP*), were significantly down-regulated in *ospil1* compared to the WT, and six *Lhcb* genes (*Lhcb1*–*6*) were severely down-regulated (Fig. 4A). In addition, *OsGLK1* and *OsGLK2*, encoding a pair of GOLDEN2-LIKE (GLK) TFs, were down-regulated in *ospil1* (Fig. 4B). The down-regulation of two *OsGLKs* was also confirmed by RT-qPCR analysis (see Supplementary Fig. S9A, B); this analysis also showed that the expression levels of these genes were higher in *OsPIL1*-OX (Fig. S9C, D). In arabidopsis, two GLK TFs (AtGLK1 and AtGLK2) directly up-regulate the expression of several genes encoding Chl-binding photosystem subunits and Chl biosynthetic enzymes (Waters *et al.*, 2009), suggesting that the down-regulation of *OsGLK* genes in *ospil1* contributes to its pale-green phenotype.

To examine whether OsPIL1 directly activates the expression of the two *OsGLK* genes, we performed a ChIP assay. We searched for the G-box (CACGTG) motif in the promoter region (–2000 bp) of *OsGLK1* and found a single motif near –1500 bp. Although the *OsGLK2* promoter does not contain a G-box motif, we found an E-box motif (CACATG; another binding motif for PIF TFs) near –1500 bp of the promoter region (Fig. 4C). ChIP assays revealed that OsPIL1 strongly binds to the promoter fragments of both *GLK* genes containing the PIF binding motifs, G- and E-boxes (Fig. 4D). Furthermore, we performed a transactivation assay using rice protoplasts to confirm that OsPIL1 acts as a transcriptional activator of the *OsGLK* genes, finding that both *OsGLK1* promoter-directed and *OsGLK2* promoter-directed GUS activity increased in the presence of *OsPIL1* expression (Fig. 4E). These results indicate that in addition to *OsPORB* and *OsCAO1*, *OsGLK1* and *OsGLK2* are also up-regulated by OsPIL1 *in vivo*.

### *OsGLK1 and OsGLK2 directly activate* OsPORB, OsCAO1, *and* LHC *genes*

**Fig. 5.**
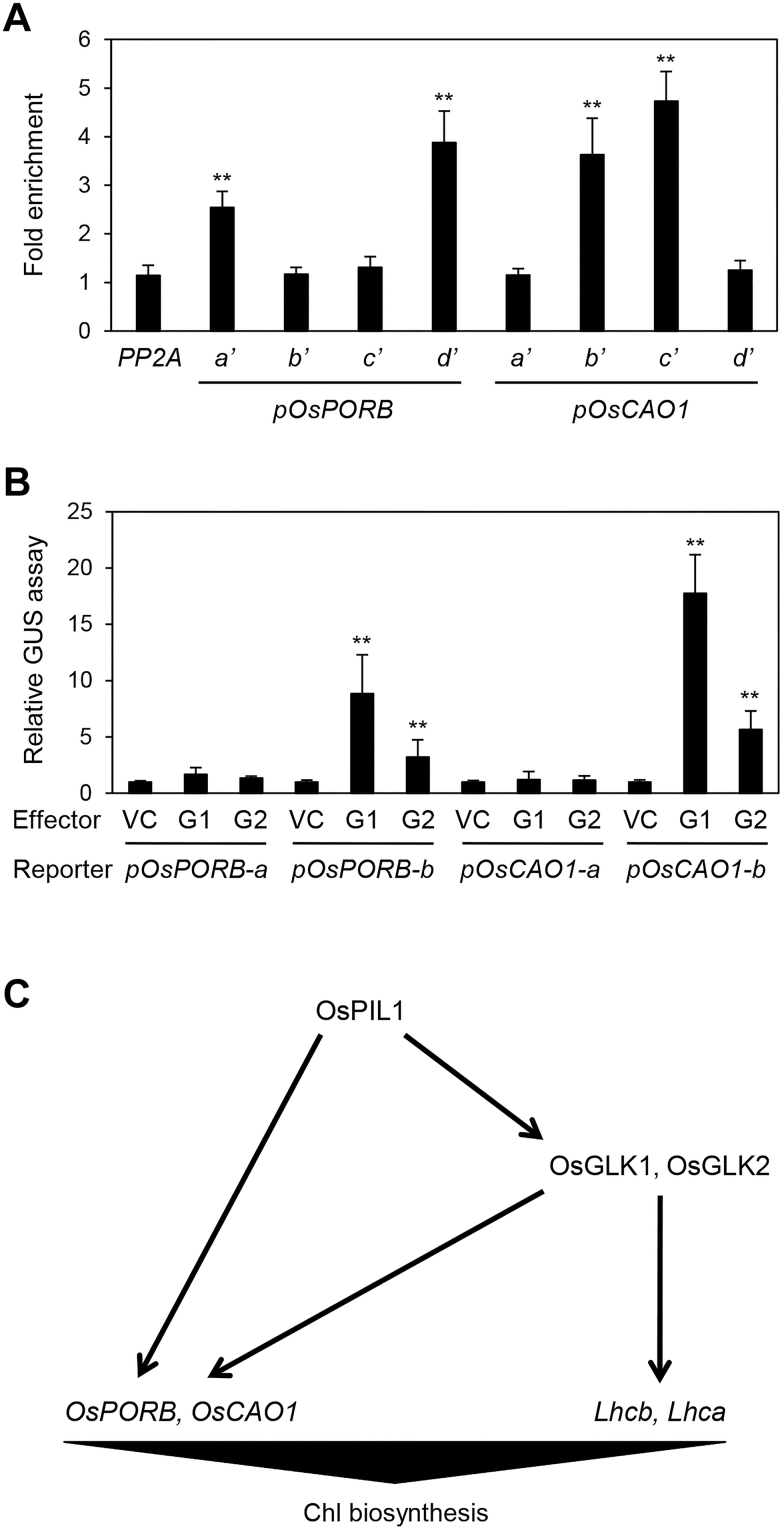
OsGLK1 and OsGLK2 also up-regulate *OsPORB* and *OsCAO1* transcription. (A) Binding of OsGLK1 and OsGLK2 to the promoter regions of *OsPORB* and *OsCAO1 in planta* examined by ChIP assays. *OsGLK1-GFP* or *OsGLK2-GFP* was transiently expressed in protoplasts isolated from 10-d-old WT seedlings. Fold-enrichment of the promoter fragments was measured by immunoprecipitation with an anti-GFP antibody (see Methods). *PP2A* was used as a negative control. (B) Transactivation of *OsPORB* and *OsCAO1* by OsGLK1 and OsGLK2. The protoplasts were co-transfected with 5 μl of effector plasmid containing *35S:OsGLK1-GFP* or *35S:OsGLK2-GFP* and 3 μl of reporter plasmids containing *pOsPORB-a::GUS*, *pOsPORB-b::GUS*, *pOsCAO1-a::GUS*, and *pOsCAO1-b::GUS*. Empty vector was used as a vector control for the effector. Significant differences were determined by Student’s *t*-test (** *P*<0.01). (C) Working model of OsPIL1-mediated up-regulation of Chl biosynthetic genes. OsPIL1 directly up-regulates the expression of *OsPORB* and *OsCAO1* by forming trifurcate feed-forward loops involving OsGLK1 and OsGLK2. Arrows indicate direct up-regulation.

In arabidopsis, two GLK TFs (GLK1 and GLK2) bind to the promoter regions of various photosynthetic and Chl biosynthetic genes, including *PORB* and *CAO1*, and up-regulate their transcription (Waters *et al.*, 2009). GLK TFs can bind to CCAATC as well as the G-box motif, CACGTG. However, these sequences are not present in the promoter regions (–2000 bp) of *OsPORB* or *OsCAO1*, although they contain a few G-box motifs (Fig. 3B). Therefore, we used ChIP assays to examine whether OsGLKs directly interact with the promoters of *OsPORB* and *OsCAO1*; these assays revealed that OsGLK1 and OsGLK2 bind to the promoter regions that contain the G-box motif (Fig. 5A). Next, we performed a transactivation assay using rice leaf protoplasts, and found that *pOsPORB-b*-directed and *pOsCAO1-b*-directed GUS activity was strongly enhanced in the presence of *OsGLK1* and *OsGLK2* expression compared with the vector control (Fig. 5B). These results indicate that *OsPORB* and *OsCAO1* are up-regulated by both OsPIL1 and OsGLK, forming trifurcate feed-forward loops for the up-regulation of Chl biosynthesis (Fig. 5C).

We further examined whether the promoters of the rice *Lhcb* and *Lhca* genes contain the GLK binding motif (CACGTG or CCAATC). Our bioinformatic analysis revealed that the promoter regions of five *Lhcb* genes (*Lhcb1*, *Lhcb2*, *Lhcb4*, *Lhcb5*, and *Lhcb6*) and five *Lhca* genes (*Lcha1*, *Lhca2*, *Lhca3*, *Lhca5*, and *Lhca6*) contain one or both motifs (see Supplementary Fig. S10A). ChIP assays showed that both OsGLK1 and OsGLK2 bind to the promoters of the four *Lhcb* genes (*Lhcb1*, *Lhcb2*, *Lhcb4*, and *Lhcb6*) and three *Lhca* genes (*Lhca1*, *Lhca2*, and *Lhca3*) (Fig. S10B). These results indicate that like arabidopsis GLKs, these two OsGLK TFs activate the expression of genes for not only Chl synthesis enzymes (*OsPORB* and *OsCAO1*) but also *Lhcb* and *Lhca* genes responsible for the accumulation of Chl and the photosystem apparatus in developing leaves.

## Discussion

### OsPIL1 TF up-regulates the expression of Chl biosynthetic and photosynthetic genes

In rice, OsPIL1 is the closest homolog of arabidopsis PIF4 among the OsPILs (Nakamura *et al.*, 2007). Arabidopsis PIF4 is involved in various biological processes, such as phytohormone synthesis, light responses, shoot elongation, flowering, circadian rhythms, stress responses, and leaf senescence (Leivar and Quail, 2011; Kumar *et al.*, 2012; Sakuraba *et al.*, 2014). Therefore, OsPIL1 probably acts as a pivotal regulator in various biological processes or signaling pathways. Todaka *et al.* (2012) described the phenotype of *OsPIL1*-OX in detail: *OsPIL1*-OX plants significantly increase in height throughout their development because of internode elongation. By transcriptome analysis, Todaka *et al.* (2012) found that *OsPIL1*-OX up-regulates cell wall synthesis-related genes, which probably promotes cell elongation of internodes. Furthermore, they found that *OsPIL1* expression was severely down-regulated in water-deficit conditions, and the set of drought stress-responsive genes were differentially expressed in *OsPIL1*-OX, strongly suggesting that the reduction of plant height under drought stress conditions is closely associated with down-regulation of *OsPIL1*.

Here, we found that OsPIL1 functions to promote Chl synthesis in leaves. Yeast one-hybrid and ChIP assays revealed that the OsPIL1 directly binds to the promoters of *OsPORB* and *OsCAO1*, both of which contain the PIF binding G-box motif, CACGTG (Fig. 3C, D). Transactivation assays further revealed that OsPIL1 activates the expression of *OsPORB* and *OsCAO1* (Fig. 3E). The physiological function of OsPORB has been determined by investigating its knockout mutant, *faded green leaf* (*fgl*); in both the paddy field and growth chamber conditions, the *fgl* mutant produced pale-green leaves with necrotic spots in the tip regions (Sakuraba *et al.*, 2013). In addition, several Chl biosynthetic genes (*OsHEMA*, *OsCHLH*, and *OsCAO1*) and photosynthesis-associated genes (*Lhcb1* and *Lhcb4*) are down-regulated in *fgl* (Sakuraba *et al.*, 2013), probably through retrograde signaling from the chloroplast to the nucleus. Thus, it is highly possible that the down-regulation of *OsPORB* partially contributes to the reduced expression levels of other Chl biosynthetic and photosynthetic genes in the *ospil1* leaves (Figs 3 and 4). We also found that *OsPORA*, another rice *POR* homolog, was significantly down-regulated in the *ospil1* mutant (Fig. 3A and Supplementary Fig. S7C), although OsPIL1 does not bind to the promoter regions of *OsPORA in vivo* (Fig. 3D). We previously found that the *OsPORA* transcript level drastically decreased after illumination of etiolated seedlings, similar to arabidopsis and wheat *PORA* (Sakuraba *et al.*, 2013); however, the *OsPORB* transcript level was not affected by illumination. In addition, the *OsPORA* transcript level was strongly affected by high light and leaf age, while the *OsPORB* transcript was less sensitive to those conditions. In addition, the overexpression of *OsPORA* in *fgl* mutants complemented the leaf chlorosis phenotype (Kwon *et al.*, 2017). Thus, the down-regulation of *OsPORA* also contributes to the pale-green phenotype of the *ospil1* mutant. Like *fgl*, the T-DNA insertional *oscao1* knockout mutant also has a pale-green leaf phenotype (Lee *et al.*, 2005). The arabidopsis *cao* mutants are deficient in Chl *b*, as the reaction from Chl *a* to Chl *b* via 7-hydroxymethyl Chl *a* is impaired by the loss of CAO catalytic activity. As a result, the Chl *a*/*b* ratios of *cao* mutants are considerably higher than the WT (Murray and Kohorn, 1991; Falbel *et al.*, 1996; Tanaka *et al.*, 1998). In this study, we found that the Chl *a*/*b* ratio of *ospil1* was significantly higher than that of the WT (see Supplementary Fig. S2F), probably due to the down-regulation of *OsCAO1*. Rice has two *CAO* homologs, *OsCAO1* and *OsCAO2* (Lee *et al.*, 2005). Unlike *OsCAO1*, however, the expression level of *OsCAO2* was not altered in *ospil1* (Fig. 3A). The expression patterns of *OsCAO1* and *OsCAO2* are quite different; *OsCAO1* mRNA levels increase in the light, while *OsCAO2* mRNA levels decrease (Lee *et al.*, 2005). Thus, OsCAO1 plays a major role in Chl *b* biosynthesis and photosynthetic protein accumulation in rice, because both Chl levels and the expression of photosynthetic genes increase upon light exposure (Ilag *et al.*, 1994; Teramoto *et al.*, 2002).

In addition to OsPIL1, we found that two rice GLK TFs, OsGLK1 and OsGLK2, also directly up-regulate the expression of *OsPORB* and *OsCAO1* (Fig. 5A, B). Because OsPIL1 is directly involved in up-regulating both *OsGLK1* and *OsGLK2*, OsPIL1 and OsGLK form coherent trifurcate feed-forward loops to induce the transcription of *OsPORB* and *OsCAO1* during Chl biosynthesis (Fig. 5C). The regulation of coherent feed-forward loops has been described previously (Kim *et al.*, 2009; Sakuraba *et al.*, 2015); this mechanism is thought to make pathways less prone to disruption by various environmental fluctuations. In arabidopsis, two GLK TFs, AtGLK1 and AtGLK2, directly up-regulate Chl biosynthetic genes, including *AtPORB* and *AtCAO*, which is similar to the role of OsGLK TFs. In addition, AtGLKs directly up-regulate photosynthetic genes such as *Lhcb* and *Lhca* (Waters *et al.*, 2009). In this study, we also found by ChIP assay that both OsGLK1 and OsGLK2 bind to the promoters of several *Lhcb* and *Lhca* genes (Supplementary Fig. S10). Therefore, it is possible that the down-regulation of *OsGLK* genes directly contributes to the reduced expression of *Lhc* genes in *ospil1*.

Cytokinin enhances Chl synthesis during greening of barley cotyledons (Yaronskaya *et al.*, 2006) and also delays the onset of leaf senescence (Romanko *et al.*, 1969). By contrast, other phytohormones, such as abscisic acid (ABA), ethylene, jasmonic acid, and salicylic acid, promote the onset of leaf senescence and Chl catabolism (Kusaba *et al.*, 2013). In our microarray analysis, several phytohormone biosynthesis- and signaling-associated genes were differentially expressed; for example, ABA synthesis genes (*ABA2*, *NCED3*, and *NCED5*) and signaling-associated genes (*ABF1*, *ABI2*, and *ABI3*) were up-regulated (see Supplementary Fig. S6E, F). Therefore, it is possible that differential expression of the phytohormone synthesis- and signaling-associated genes affect Chl accumulation in the *ospil1* leaves.

 Collectively, these findings suggest that OsPIL1 directly or indirectly enhances the expression of Chl biosynthetic and photosynthetic genes via various regulatory cascades.

### The differences and similarities between OsPIL1 and arabidopsis PIFs

In this study, we found that *ospil1* plants had pale-green leaves in both the paddy field (Fig. 1) and in the growth chambers (see Supplementary Figs S2 and S4). However, this color-defective phenotype has not been observed in arabidopsis *pif* mutants, including *pif1*, *pif3*, *pif4*, and *pif5*; at the vegetative stage, these mutants produce normal green leaves like those of the WT, although Chl biosynthesis is strongly inhibited in de-etiolated seedlings of *pif1* upon light exposure (Huq *et al.*, 2004). Thus, the physiological roles of PIFs in Chl biosynthesis in arabidopsis and rice (at least OsPIL1) are somehow different, although OsPIL1 is phylogenetically the closest homolog of arabidopsis PIF4 (Nakamura *et al.*, 2007).

 In arabidopsis, phyB interacts with and rapidly phosphorylates PIFs, leading to ubiquitination and degradation of PIFs by the 26S proteasome system (Al-Sady *et al.*, 2006). Thus, the hypocotyl, petiole, flowering time, and leaf senescence phenotypes of arabidopsis *phyB* mutants are opposite to those of *pif* mutants (Huq and Quail, 2002; Nozue *et al.*, 2007; Kumar *et al.*, 2012; Sakuraba *et al.*, 2014). Interestingly, OsPIL1 does not interact with OsphyB (Todaka *et al.*, 2012), indicating that the stability of OsPIL1 is not regulated by OsphyB at the post-translational level. Indeed, *osphyB* knockout mutant plants are considerably shorter than the WT (Takano *et al.*, 2005), with pale-green leaves (Inagaki *et al.*, 2015), like those of the *ospil1* mutant observed in the present study (Fig. 1, and Supplementary Figs S2 and S4).

It is currently unknown if OsphyB indirectly up-regulates or down-regulates the expression of *OsPIL1*. Arabidopsis PIF4 and PIF5 directly up-regulate another *PIF* gene, *AtPIL1* (Hornitschek *et al.*, 2012). Thus, *AtPIL1* expression is up-regulated in the *phyB* mutant (Sakuraba *et al.*, 2014). A transactivation assay showed that OsphyB does not affect *OsPIL1* expression (Todaka *et al.*, 2012). However, we previously found that *OsPIL1* expression is down-regulated in *osphyB* mutants during dark-induced senescence, although this down-regulation is negligible under normal growth conditions (Piao *et al.*, 2015). Therefore, it is possible that OsphyB indirectly regulates *OsPIL1* expression positively or negatively under specific conditions, such as darkness. Further investigation of the molecular connection between OsphyB and OsPIL1 will be necessary to understand phyB-mediated red-light signaling in rice in more detail.

## Supplementary Data

Supplementary data are available at *JXB* online.

Fig. S1. Complementation of the pale-green phenotype of *ospil1*.

Fig. S2. Characterization of the pale-green phenotype of *ospil1*.

Fig. S3. TEM images showing the structures of chloroplasts and thylakoid membranes in WT and *ospil1* leaves.

Fig. S4. The *ospil1* mutant has pale-green leaves under both LD and SD conditions.

Fig. S5. Cell wall-related genes are down-regulated in *ospil1*.

Fig. S6. Expression analysis of phytohormone biosynthesis- and signaling-associated genes in *ospil1*.

Fig. S7. Chlorophyll biosynthetic gene expression is reduced in *ospil1*.

Fig. S8. Expression of chlorophyll biosynthetic genes in *OsPIL1*-OX.

Fig. S9. The expression of *OsGLK1* and *OsGLK2* in *ospil1* and *OsPIL1*-OX.

Fig. S10. OsGLK1 and OsGLK2 directly up-regulate genes encoding components of the photosystem apparatus.

Table S1. Primers used in this study.

## Supplementary Material

Supplementary Figures
