# Supplementary material for: Rice Phytochrome-Interacting Factor-Like1 (OsPIL1) is involved in the promotion of chlorophyll biosynthesis through feed-forward regulatory loops
Source: J Exp Bot. 2017 Jul 11;68(15):4103–14. doi: 10.1093/jxb/erx231 (PMC5853433; doi:10.1093/jxb/erx231)
Supplement: Supplementary Figures [file erx231_suppl_supplementary_figures.pdf]

Supplementary Data

Rice Phytochrome-Interacting Factor-Like1 (OsPIL1) is involved in the promotion of chlorophyll biosynthesis through feed-forward regulatory loops

Yasuhito Sakuraba, Eun-Young Kim, Su-Hyun Han, Weilan Piao, Gynheung An, Daisuke Todaka, Kazuko Yamaguchi-Shinozaki, and Nam-Chon Paek

Fig. S1. Complementation of the pale-green phenotype of *ospil1*.
Fig. S2. Characterization of the pale green phenotype of *ospil1*.
Fig. S3. TEM images showing the structures of chloroplasts and thylakoid membranes in WT and *ospil1* leaves.
Fig. S4. The *ospil1* mutant has pale-green leaves under both LD and SD conditions.
Fig. S5. Cell wall-related genes are downregulated in *ospil1*.
Fig. S6. Expression analysis of phytohormone biosynthesis- and signaling-associated genes in *ospil1*.
Fig. S7. Chl biosynthetic gene expression is reduced in *ospil1*.
Fig. S8. Expression of Chl biosynthetic genes in *OsPIL1*-OX.
Fig. S9. The expression of *OsGLK1* and *OsGLK2* in *ospil1* and *OsPIL1*-OX.
Fig. S10. OsGLK1 and OsGLK2 directly upregulate genes encoding components of the photosystem apparatus.
Table S1. Primers used in this study.

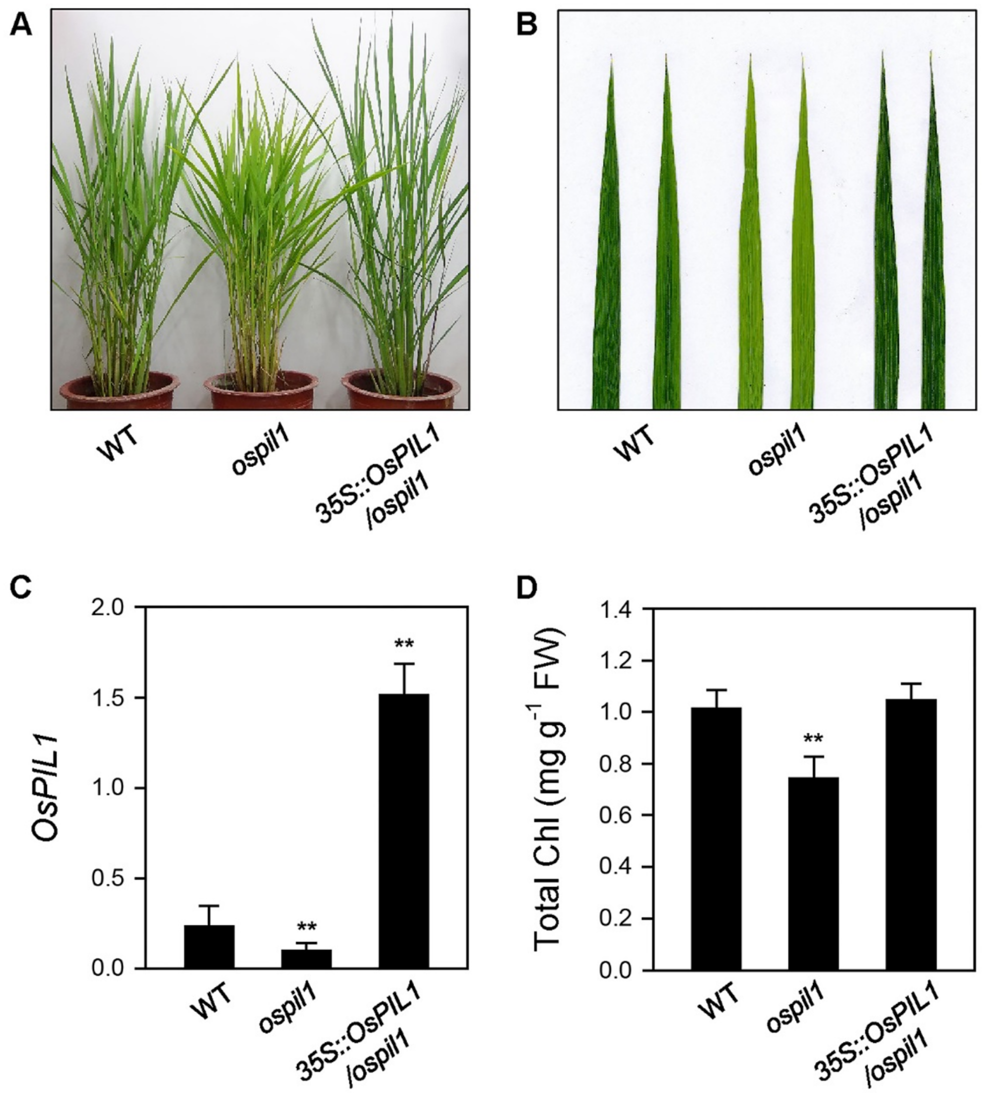

Fig. S1. Complementation of the pale-green leaf phenotype of *ospil1*.

(A, B) Phenotypes of 90 DAS plants (A) and first leaves of WT, *ospil1*, and *35S:OsPIL1/ospil1* (B). (C) *OsPIL1* expression levels in WT, *ospil1*, and *35S:OsPIL1/ospil1* plants, as measured by RT-qPCR and normalized to the transcript levels of *UBQ5*. (D) Total Chl levels were measured using the middle part of the second leaves in the main culm. (C, D) Mean and s.d. values were obtained from three biological replicates. Student's *t*-test (* $P < 0.05$, ** $P < 0.01$).

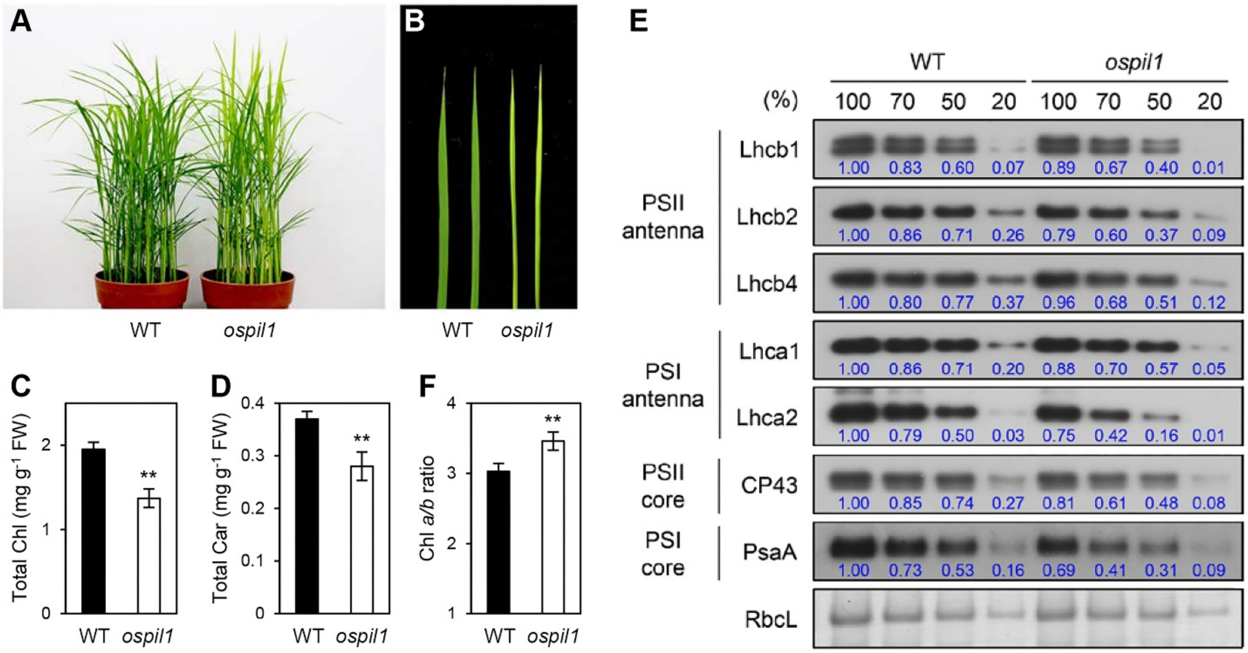

Fig. S2. Characterization of the pale green phenotype of *ospil1*.

(A, B) Phenotypes of 15-day-old WT and *ospil1* plants grown in a growth chamber (14.5 h light, 30°C/9.5 h dark, 24°C, 300 µmol m^{-2} s^{-1}) (A) and first leaves of WT and *ospil1* plants (B). (C–E) Changes in total Chl (C), total Car (D), and photosynthetic protein (E) levels and the Chl *a/b* ratio (F). (C, D, F) Pigment levels in the first leaves of 15-day-old WT and *ospil1* plants. Mean and s.d. values were obtained from ten biological replicates (n=10). Student's *t*-test (** $P < 0.01$). (D) Immunoblot analysis of photosystem proteins in the first leaves of 6-week-old WT and *ospil1* plants. Antibodies against the PSI (Lhca1, Lhca2, and PsaA) and PSII subunits (Lhcb1, Lhcb2, Lhcb4, and CP43) were used for detection. RbcL (Rubisco large subunit) protein was visualized by Coomassie Brilliant Blue staining. Numbers indicate the relative amounts of proteins normalized to WT (100%; first lane).

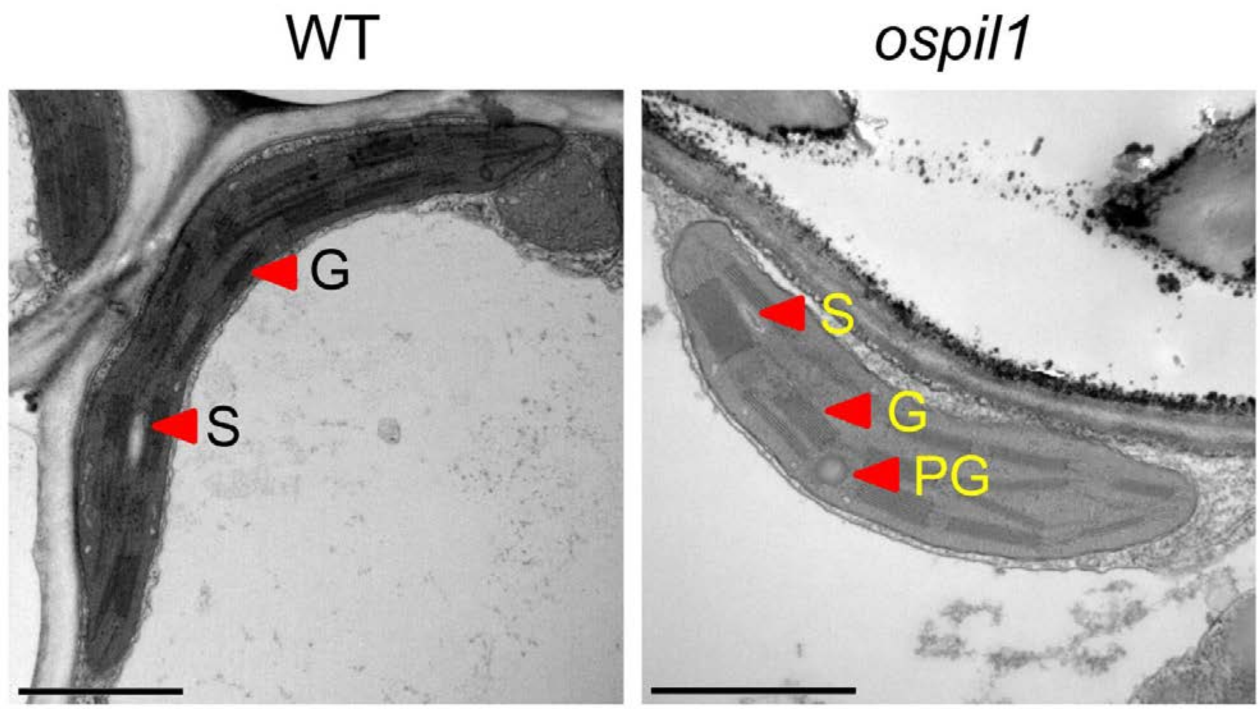

Fig. S3. TEM images showing the structures of chloroplasts and thylakoid membranes in WT and *ospil1* leaves.

Tissue samples were obtained from the first leaves of 6-week-old WT and *ospil1* plants. G, grana; PG, plastoglobule; S, starch. Bars = 1 μm.

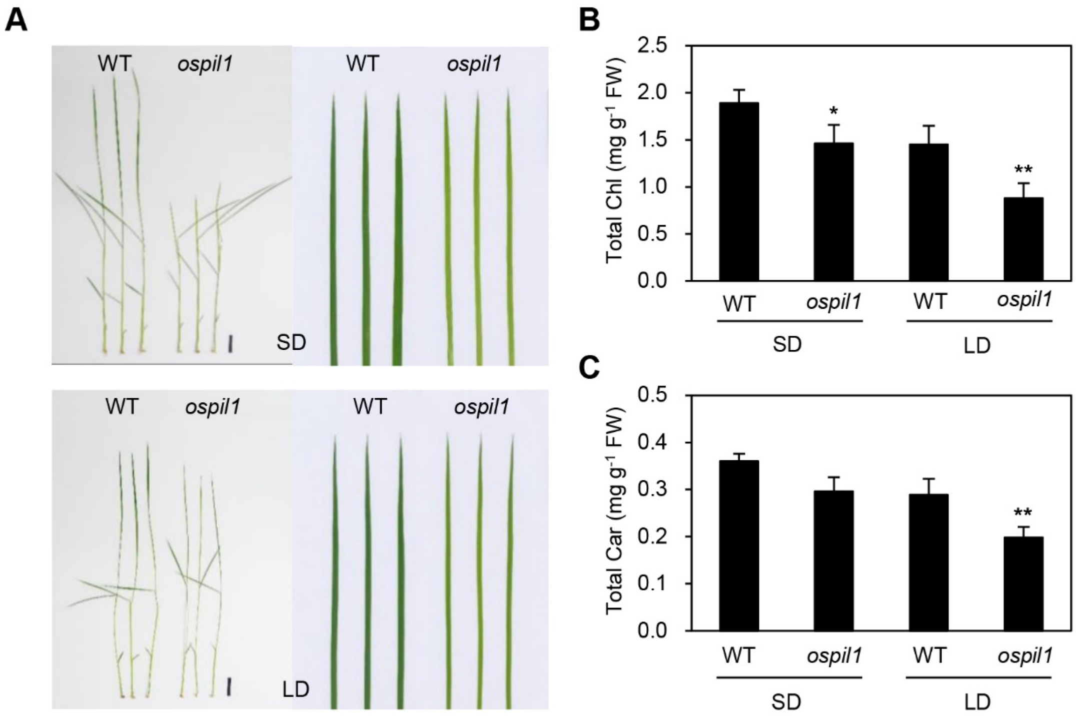

Fig. S4. The *ospil1* mutant has pale-green leaves under SD and LD conditions.

(A) Comparison of leaf color of 3-week-old WT and *ospil1* plants grown in a growth chamber under SD (10 h light, 30°C /14 h dark, 24°C; upper panel) and LD (14.5 h light, 30°C/9.5 h dark, 24°C; lower panel) conditions. (B, C) Total Chl (B) and Car (C) levels were measured using the middle parts of second leaves of WT and *ospil1* plants grown in a growth chamber under SD and LD conditions. Student's *t*-test (* $P < 0.05$, ** $P < 0.01$).

Gene encoding	Gene Index	*ospil1* / WT
α-Expansin *OsEXPA5*	Os02g0744200	
α-Expansin *OsEXPA2*	Os01g0823100	**
α-Expansin *OsEXPA21*	Os03g0377100	*
α-Expansin *OsEXPA4*	Os05g0477600	**
α-Expansin *OsEXPA19*	Os03g0156000	**
α-Expansin *OsEXPA5*	Os01g0274500	
β-Expansin 5	Os04g0552200	*
β-Expansin	Os02g0658800	**
β-Expansin	Os10g0555900	**
Cellulose synthase-1	Os05g0176100	**
Cellulose synthase-4	Os07g0208500	
Cellulose synthase-7	Os03g0837100	
Cellulose synthase-8	Os07g0252400	**
CSLF6 (fragment)	Os08g0160500	**
EXPANSIN S2	Os04g0228400	*
EXPANSIN S1	Os03g0336400	**
Glycoside hydrolase	Os09g0494200	
LysM-domain GPI-anchored protein 1 precursor	Os09g0452200	**
Pectin methylesterase isoform-α (fragment)	Os01g0312500	**
Pectinesterase	Os08g0450100	**
Pectinesterase	Os07g0675200	*
Pectinesterase	Os06g0193200	**

(Log_2 FC) -1.0 -0.8 -0.6 -0.4 -0.2 0

Fig. S5. Cell wall-associated genes are downregulated in *ospil1*.

The relative expression levels of cell wall-associated genes (*ospil1*/WT) are listed. These genes were previously shown to be significantly upregulated in *OsPIL1*-OX plants (Todaka et al. 2013). The relative expression levels of genes in *ospil1* were normalized to those of WT. Asterisks in each column indicate significant difference between WT and *ospil1* plants (* $P < 0.05$, ** $P < 0.01$).

A

Gene	Gene Index	*ospil1*/WT
GA2OX3	Os01g0757200	**
GA2OX6	Os04g0522500	**
GA2OX7	Os01g0209700	*
GA2OX8	Os05g0560900	**
GA3OX1	Os05g0178100	
GA30X2	Os01g0177400	**

B

OsDELLA	Os03g0707600	

C

ACS1	Os03g0727600	
ACS2	Os04g0578000	**
ACS6	Os06g0130400	*
ACS9	Os04g0310800	

D

EIN2	Os07g0155600	
EIN3	Os03g0324200	**
ERF1	Os04g0546800	
ERF2	Os01g0752500	
ERF3	Os01g0797600	
ERF5	Os07g0410300	
ERF7	Os06g0166400	**
ERF8	Os04g0648900	
ERF9	Os03g0263000	

E

ABA1	Os04g0448900	
ABA2	Os03g0810800	**
ABA3	Os06g0670000	
ABA4	Os01g0128300	
CYP707A1	Os02g0703600	
CYP707A2	Os09g0457100	
CYP707A3	Os08g0472800	**
NCED2	Os03g0645900	
NCED3	Os12g0617400	**
NCED5	Os07g0154100	**

F

ABF1	Os01g0859300	**
ABF2	Os06g0211200	
ABI2	Os01g0583100	**
ABI3	Os01g0911700	**
ABI4	Os05g0351200	
ABI5	Os09g0456200	

G

Gene	Gene	*ospil1*/WT
OsNIT	Os02g0635200	
OsAAO	Os03g0790900	
YUCCA4	Os01g0224700	
YUCCA6	Os07g0437000	
YUCCA10	Os01g0274100	
YUCCA11	Os12g0189500	

H

OsIAA1	Os01g0178500	
OsIAA2	Os02g0805100	*
OsIAA5	Os02g0817600	
OsIAA20	Os06g0166500	**
OsIAA3	Os01g0231000	
OsARF1	Os11g0523800	
OsARF2	Os01g0927600	
OsARF3	Os01g0753500	
OsARF5	Os02g0141100	**
OsARF8	Os02g0628600	
OsARF9	Os04g0442000	
OsARF12	Os04g0671900	**

I

CKX2	Os01g0197700	
CKX3	Os10g0483500	*
CKX4	Os01g0940000	**
CKX5	Os01g0775400	

J

OsRR1	Os04g0442300	
OsRR2	Os02g0557800	
OsRR3	Os02g0830200	**
OsRR4	Os01g0952500	**
OsRR5	Os04g0524300	
OsRR6	Os04g0673300	
AHK3	Os01g0923700	
AHK5	Os10g0362300	

K

OsCPD1	Os11g0143200	
OsCPD2	Os12g0139300	
DWF1	Os10g0397400	*

L

OsBZR1	Os07g0580500	
OsBRI1	Os01g0718300	
OsBIN2	Os05g0207500	

M

Gene	Gene Index	*ospil1*/WT
SLB2	Os01g0701400	
MAX3	Os04g0550600	
MAX4	Os01g0566500	**

N

OsDAD1	Os04g0397000	
OsDGL	Os07g0209000	
OsLOX2	Os03g0179900	
OsAOS	Os03g0225900	
OsOPR1	Os06g0216300	
OsJAR1	Os05g0586200	

O

OsCOI1a	Os01g0853400	
OsCOI1b	Os05g0449500	
OsJAZ1	Os10g0392400	**
OsJAZ7	Os07g0615200	
OsJAZ6	Os03g0402800	
OsJAZ10	Os03g0181100	
OsMYC2	Os10g0575000	

P

PAL1	Os02g0626100	**
PAL2	Os02g0626400	**
S3H	Os04g0581100	**

Q

NPR1	Os01g0194300	
NPR2	Os01g0767900	
NPR3	Os03g0667100	
NPR4	Os01g0837000	*

(Log_2 FC) -0.6 -0.4 -0.2 0.1 0.2 0.3

Fig. S6. Expression analysis of phytohormone biosynthesis- and signaling-associated genes in *ospil1*.

Relative expression (*ospil1*/WT) of genes involved in gibberellic acid biosynthesis (A), gibberellic acid signaling (B), ethylene biosynthesis (C), ethylene signaling (D), abscisic acid biosynthesis (E), abscisic acid signaling (F), auxin biosynthesis (G), auxin signaling (H), cytokinin biosynthesis (I), cytokinin signaling (J), brassinosteroid biosynthesis (K), brassinosteroid signaling (L), strigolactone biosynthesis (M), jasmonic acid biosynthesis (N), jasmonic acid signaling (O), salicylic acid biosynthesis (P), and salicylic acid signaling (Q). Relative expression levels of genes in *ospil1* were normalized to those of WT. Asterisks in each column indicate significant difference between WT and *ospil1* plants (* $P < 0.05$, ** $P < 0.01$).

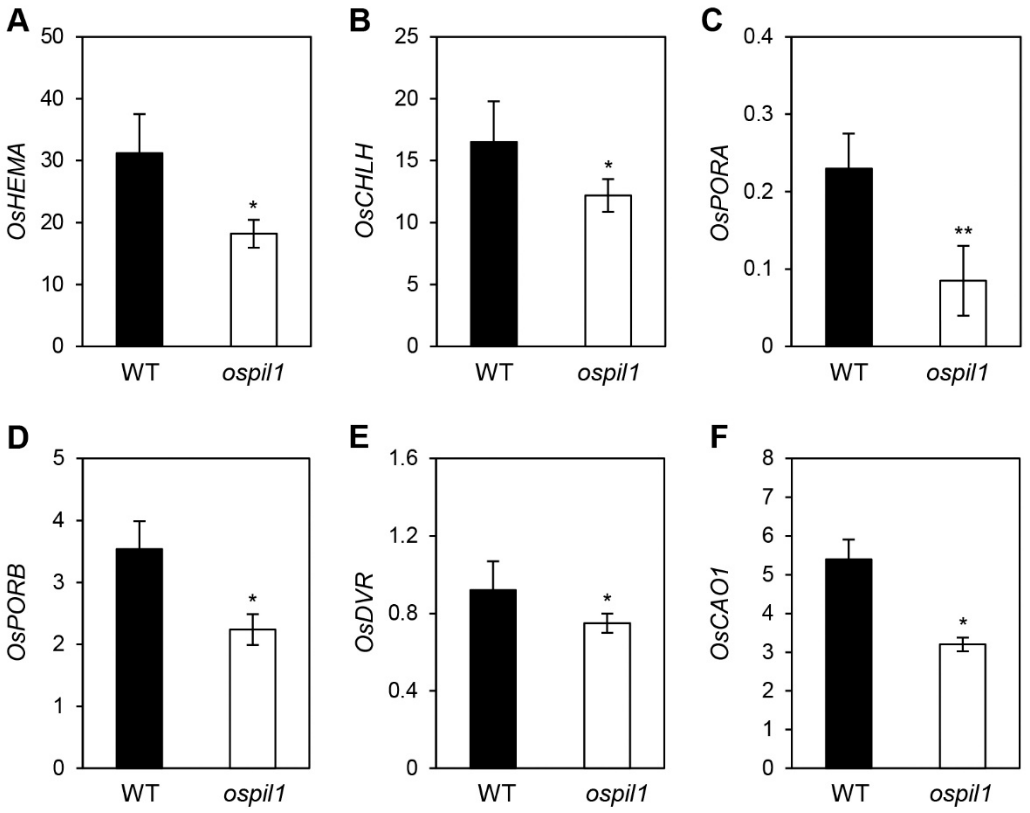

Fig. S7. Expression of Chl biosynthetic genes in *OsPIL1*-OX.

Total RNA was extracted from the first leaves of 3-week-old WT and *OsPIL1*-OX plants grown in a growth chamber under LD conditions. Relative transcript levels of *OsHEMA* (A), *OsCHLH* (B), *OsPORA* (C), *OsPORB* (D), *OsDVR* (E), and *OsCAO1* (F) were obtained by normalizing to the transcript level of *GAPDH*. Mean and s.d. values were obtained from three biological replicates. Student's *t*-test (* $P < 0.05$).

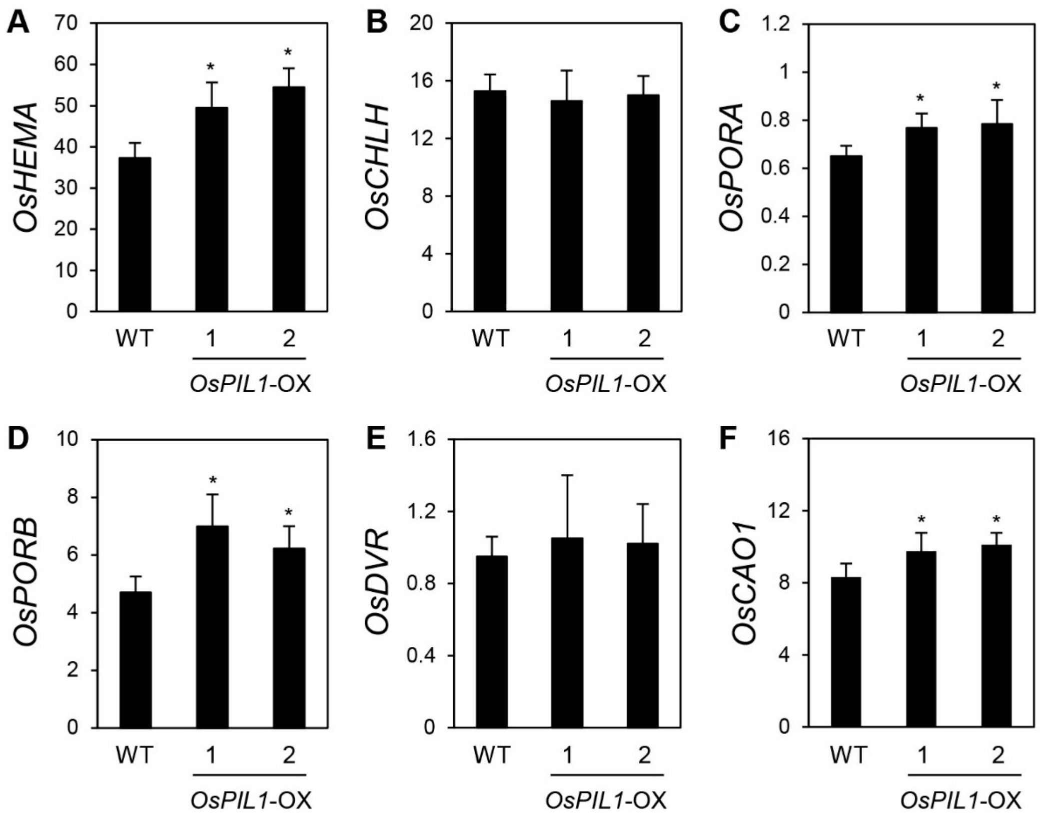

Fig. S8. Expression of Chl biosynthetic genes in *OsPIL1*-OX.

Total RNA was extracted from the first leaves of 3-week-old WT and *OsPIL1*-OX plants grown in a growth chamber under LD conditions. Relative transcript levels of *OsHEMA* (A), *OsCHLH* (B), *OsPORA* (C), *OsPORB* (D), *OsDVR* (E), and *OsCAO1* (F) were obtained by normalizing to the transcript level of *GAPDH*. Mean and s.d. values were obtained from three biological replicates. Student's *t*-test (* $P < 0.05$).

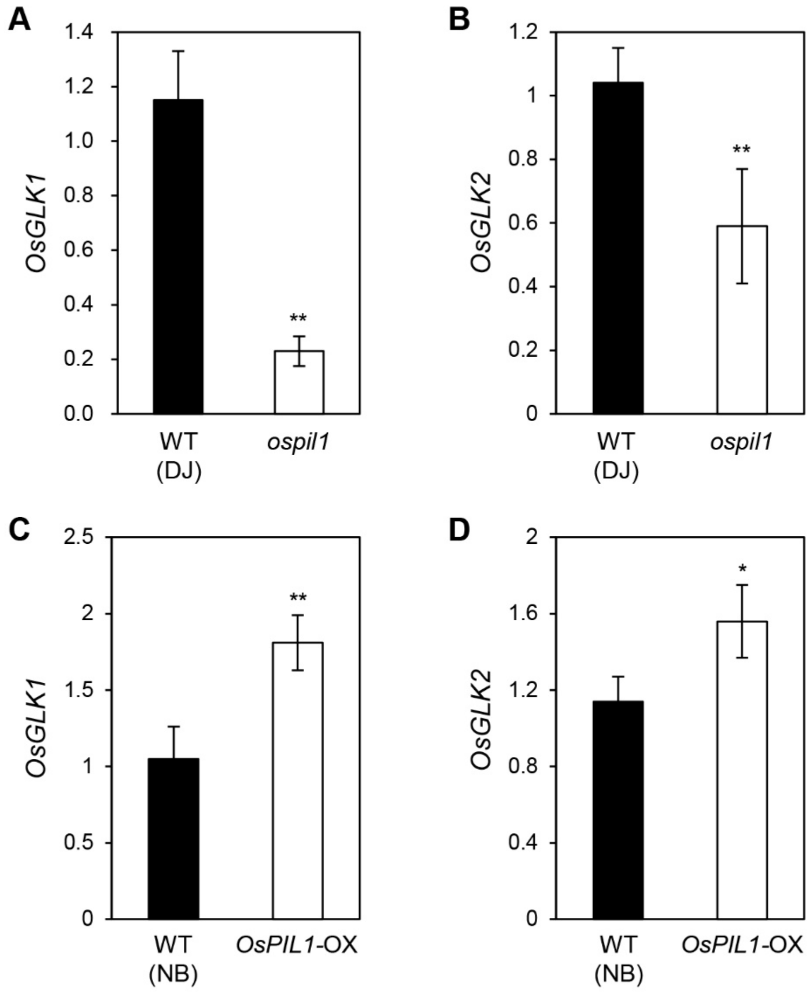

Fig. S9. The expression of *OsGLK1* and *OsGLK2* in *ospil1* and *OsPIL1*-OX.

Total RNA was extracted from the first leaves of 3-week-old WT (Dongjin WT for *ospil1* and Nipponbare for *OsPIL1*-OX), *ospil1*, *OsPIL1*-OX plants grown in a growth chambers under LD conditions. Relative expression levels of *OsGLK1* (A, C) and *OsGLK2* (B, D) were examined in *ospil1* (A, B) and *OsPIL1*-OX (C, D) respectively, and were determined by normalizing to the transcript level of *GAPDH*. Mean and s.d. values were obtained from three biological replicates. Student's *t*-test (** $P < 0.01$).

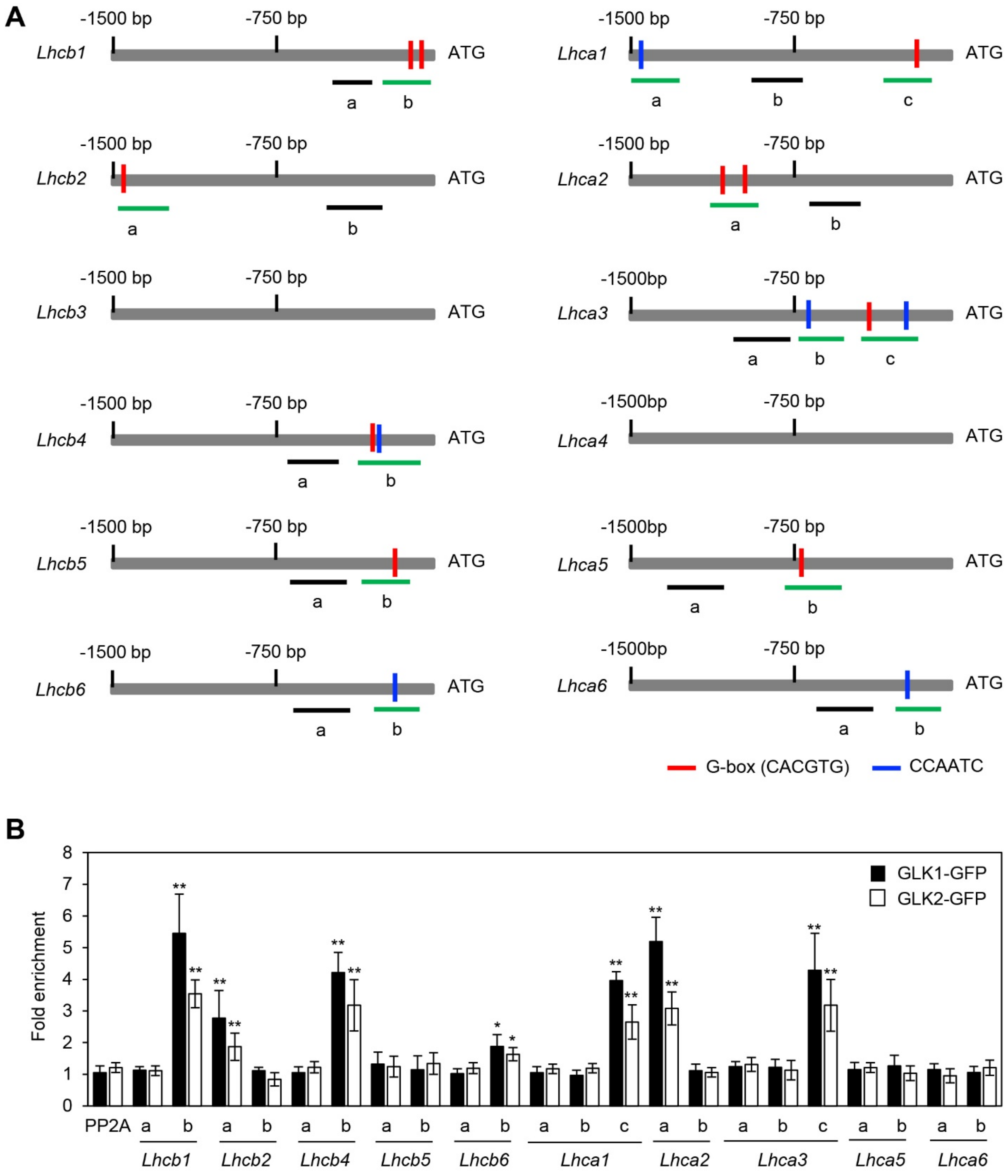

Fig. S10. OsGLK1 and OsGLK2 directly upregulate photosystem apparatus genes.

(A) Positions of the OsGLK binding motifs CCAATC (red vertical bar) and G-box (CACGTG, blue vertical bar) in the promoters of six *Lhcb* genes (*Lhcb1*, *Lhcb2*, *Lhcb3*, *Lhcb4*, *Lhcb5*, and *Lhcb6*) and six *Lhca* genes (*Lhca1*, *Lhca2*, *Lhca3*, *Lhca4*, *Lhca5*, and *Lhca6*). The promoter

fragments containing or lacking the GLK binding motif (green horizontal bars) are indicated by green and black horizontal bars, respectively. (B) (A) OsGLK1 and OsGLK2 binding affinity to the promoter regions of *Lhcb* and *Lhca* genes *in planta* examined by ChIP assays. OsGLK1-GFP or OsGLK2-GFP was transiently expressed in protoplasts isolated from 10-day-old WT seedlings. Fold enrichment of the promoter fragments was measured by immunoprecipitation with an anti-GFP antibody (see Methods). *PP2A* was used as a negative control. Student's *t*-test (* $P < 0.05$, ** $P < 0.01$).

Table S1. Primers used in this study.

	Forward primer (5’→3’)	Reverse primer (5’→3’)
A. Primers for verification of transgenic plants		
PFG 4A-03590.R	ATTGGAGCAAATGCACACAG	ACAGAGCCCTAAGCTTGCAG
B. Primers used for gene cloning		
OsPIL1	ATGGCGATTTGCAGCACGGACAAC	CTAAATTCCATCAGAGGTTGGTGG
OsPIL1-GAD (Y1H)	GGATCCTAATGGCGATTTGCAGCACGGACAA	CTCGAGCTAAATTCCATCAGAGGTTGGTGG
proOsPORA -a (Y1H)	GAATTCGGATCAGGTCATTTTGGTCAAAC	CTCGAGACACGTCAATATATTAGCAAGGA
proOsPORA -b (Y1H)	GAATTCTTTACCTGACATTTTGGACAGAG	CTCGAGTGGTGTGAGTGTTTGAGTTTG
proOsPORB -a (Y1H)	GAATTCGAAGCTGGGGAAGAAGCTAGCG	CTCGAGATGTAAGCACGATGTAATTAG
proOsPORB -b (Y1H)	GAATTCTTTCGAAACACACGCCTGCTTTTTTTC	CTCGAGCGGAGATTTTTCTCTCTTTTTTTTC
proOsCAO1-a (Y1H)	GAATTCGGAGAGAGTACTGCGTATGAAG	CTCGAGGGATGAAGTAGAATTTTTCGGC
proOsCAO1-b (Y1H)	GAATTCCATTTGTTCAGTTGTACCATATTC	GTCGACGGATGCTACGATCGAACAGTGC
C. Primers used for qRT-PCR		
OsPIL1	ATGCCAAATCACATCCCTCTAA	GCTTGCCTCTCTCAGTTGAA
OsHEMA	CGCTATTTCTGATGCTATGGGT	TCTTGGGTGATGATTGTTTGG
OsCHLH	AACTGGATGAGCCAGAAGAGA	AAATGCAAAAGACTTGCGACT
OsPORA	ATGGCTCTCCAAGTTCAGG	CTTCTGGCTCACGCTAAGGAA
OsPORB	AGCTCCTCGCCGACCTCA	TCCCCCAGGTTCGCCTT
OsDVR	GATCCATACCCGATCGACAT	CGAGAGACATCCGGTAGAGC
OsCAO1	AGCCCAGGTTCATCAAGGT	TGATCACCCTCTCGAAGAACT
OsGLK1	ACACATGATTGCGAGAGAGG	GAGGAGGAGGGAAGCCAAT
OsGLK2	AGCAGCTCGGGATAGACAAG	CTCGCCATCAGATGTTTCCT
OsUBQ5	ACCACTTCGACCGCCACTACT	ACGCCTAAGCCTGCTGGTT
OsGAPDH	AAGCCAGCATCCTATGATCAGATT	CGTAACCCAGAATACCCTTGAGTTT
D. Primers used for ChIP-qPCR		
OsPORA-a'	AGTTGTGACCAAGTAAGCTGAGCA	TTACTGGTTAATCTGACCAGTAACTTGA
OsPORA-b'	GGTCAAGTTACTGGTCAGATTAACCA	TTCATCGCCTCTTCTTGGTGT
OsPORA-c'	ACACCAAGAAGAGGCGATGAA	TAACATATGGTGACGTGGCGTT
OsPORA-d'	AACGCCACGTCACCATATGTTA	TGGTGTGAGTGTTTGAGTTTGGA
OsPORB-a'	AATGAAGCCTCGTCCTCTCTTTTAC	ACAAGTCGAGCAGCAGGTCGT
OsPORB-b'	AATCTATCGCATGATGTATAGGCAGTC	CATGACCCTTAAAAGTGATCGAAA
OsPORB-c'	TTTCGATCACTTTTAAGGGTCATG	GTTTCTCGTCGTTTTTATCTCCTCT
OsPORB-d'	CAGCGCACATAGAGGAGATAAAAA	CGGAGATTTTTCTCTCTTTTTTTTCC
OsCAO1-a'	GGGTTATAAAATGAGGAAGTATCGTCC	TGACTTACATTGTGAAACGGATGAA
OsCAO1-b'	ACTTCATCCGTTTCACAATGTAAGT	AATAATTTTCGCGGGCGAG

OsCAO1-c'	TCGCCCGCGAAAATTATTATT	GCCCTGAACCCTTATCTGCT
OsCAO1-d'	GCGAGCAGATAAGGGTTCAG	GGATGCTACGATCGAACAGTG
OsGLK1-a'	CCTCAATCATCTCAATAGATGGTTTT	TTTACTTGAGCAATTTTATCAAGTTTCAG
OsGLK1-b'	GATATAGCAGAACCAAGGATGGTTTG	GATAAGGTTTACTTGGTTCAGGAGCTC
OsGLK1-c'	GTCTACTTACAAATCCTCTGCAGTTTG	AAGTGCAGCTTGGTCGCTTCT
OsGLK2-a'	CTAATTACATGTGGTAAGTCCATTAAA	GGTACCCACGTATGTAGGTGTGTAC
OsGLK2-b'	AATACAAGTGCCCGCGCATATA	ACGGATGCAACTTGCCAAA
OsGLK2-c'	TAATCTCGAGGAGTTTTGTGGTC	TGCTGGATTTAGAGGCAAAGCT
